# Approaching the low optical loss limit of plasmonics using potassium

**DOI:** 10.1038/s41377-026-02400-8

**Published:** 2026-07-21

**Authors:** Yi Zhang, Yuhan Yang, Jie Liang, Shizhuang Wang, Yuhong Shi, Pengcheng Yao, Hanyu Fu, Jacob B. Khurgin, Fengrui Hu, Jia Zhu, Shining Zhu, Lin Zhou

**Affiliations:** 1 National Laboratory of Solid State Microstructures, College of Engineering and Applied Sciences, Key Laboratory of Intelligent Optical Sensing and Manipulation, Nanjing University, Nanjing, 210093 China; 2 School of Information and Electronic Engineering (Sussex Artificial Intelligence Institute), Zhejiang Gongshang University, Hangzhou, 310018 China; 3 Department of Electrical and Computer Engineering, Whiting School of Engineering, Johns Hopkins University, Baltimore, MD 21218 USA

**Keywords:** Nanophotonics and plasmonics, Metamaterials

## Abstract

Plasmonics enables the miniaturization of photonic devices beyond the optical diffraction limit, yet its potential is hindered by inherently large ohmic losses. Hence, it is prudent to explore low-loss alternatives to the current mainstay of plasmonics—the noble metals. In this work, we demonstrate the potential of potassium as a plasmonic material with intrinsically low losses in the optical region. The ultra-flat, high-quality potassium film, fabricated via a rapid slipping-assisted oxide-free crystallization process, achieves measured optical damping rate down to 2.27 meV, with a measured imaginary permittivity of ~0.1 across the entire visible to near-infrared range (400–2000 nm). Near-field optical spectroscopic measurements further confirmed the reduced losses by revealing deeply subwavelength confinement of optical modes. This result overcomes the loss-confinement tradeoff existing in state-of-the-art plasmonic materials and devices, establishing a new platform for exploring extreme light–matter interactions in a variety of plasmonic systems.

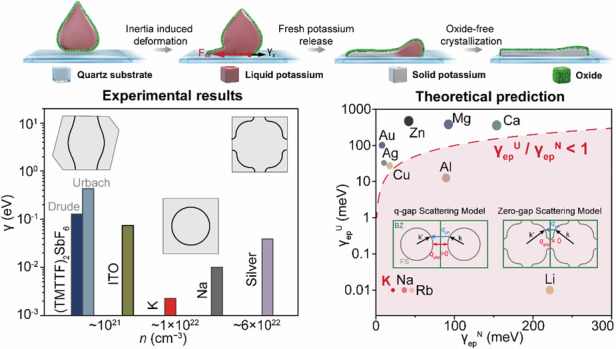

## Abstract (web summary)

The potassium film, fabricated via a rapid slipping-assisted oxide-free crystallization process, achieves the lowest optical loss measured in metals to date, while simultaneously maintaining deeply subwavelength confinement of optical mode.

## Introduction

Plasmonics enables the manipulation of electromagnetic fields beyond the optical diffraction limit and has been widely explored for enhanced light–matter interactions^1–4^, super-resolution imaging^5–8^, and low-threshold nanolasers^9–13^. However, practical plasmonic devices composed of noble metals^14–18^ and other state-of-the-art materials^19^ are fundamentally constrained by considerable ohmic damping as well as a severe trade-off between optical losses and field confinement.

Alkali metals, especially potassium, are known as good examples of near-free-electron behavior as noble metals, and have excited intensive modern experimental studies dating back to the 1930s^15^ and revived in the 1960s^16^. However, the low melting points and high chemical reactivity result in huge surface roughness and severe adsorption contamination, even under ultrahigh vacuum (UHV) deposition conditions. Furthermore, most in-situ integrated spectroscopic ellipsometry (SE) exhibits low accuracy as the targeting materials are of low loss or high reflectance^16,17^, measurements based on which are mostly limited in the ultraviolet (UV) and visible wavelengths below the interband absorption opening (*λ* < 600 nm) for UV-transparent or photoemission materials^18^. Although few groups have extended the optical measurements to the near or mid infrared^18–20^, considerable deviation from the standard Drude model (off-straight-line for optical mass, frequency-dependent damping rate, anomalous absorption, etc.) as well as sample-to-sample inconsistency, unraveling the poor reliability and thus hindering the development of alkali-metal plasmonics for more than half a century.

Recently, intriguing thermo-assisted methods beyond UHV deposition have been introduced for high-quality sodium nanostructures^21,22^, which enable marginal optical loss reduction (with a damping rate ~ one half of silver), and especially stable high-performing devices, arousing broad interest in photophysics and nanophotonics^23,24^. Similar embossing attempts in potassium-related systems have yet to show much relevance to low-loss plasmonic materials due to higher chemical reactivity and stability issues.

Despite the lack of reliable experimental material data for potassium, the theoretical inputs have never stopped since the earliest works on DC conductivity investigations. More theoretical endeavors are searching for low-loss plasmonic materials in the optical, especially near-infrared range^25,26^. It is noteworthy that, although the basic theory by scattering process for optical loss reduction is known for a century, the quantitative efforts are mainly based on energy-resolved optical transition predicted by Khurgin and coworkers^26^, which ignites the emergence of low-loss hypergap transparent conductors very recently^19^. However, the indiscriminate bandwidth reduction and bandgap enlargement inevitably deteriorate the optical confinement.

In this work, by quantitatively evaluating the plasmonic losses based on the momentum-gap-resolved scattering model, we theoretically identify potassium (K) as the ideal plasmonic metal approaching the low-loss limit in the optical frequency range. In the experiment, high-quality and low-loss potassium is prepared via ultrafast slipping-assisted oxide-free crystallization (SOC) and measured by analytical SE measurements, enabling record-low optical loss of intrinsic potassium as well as deep-subwavelength field concentration, overcoming the conventional loss-confinement trade-off in existing plasmonic systems.

## Results

### Theoretical analysis

**Fig. 1:**
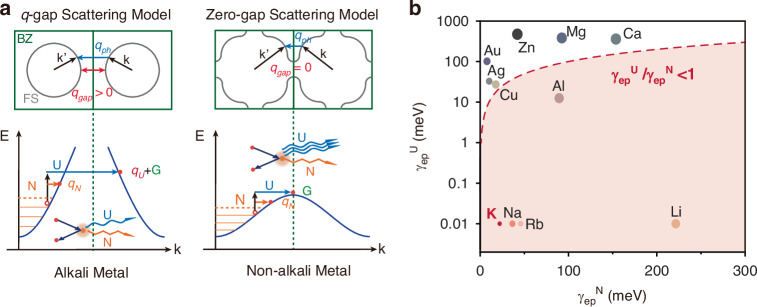
Theoretical prediction on record-low plasmonic loss of potassium metal. **a** The electron Umklapp scattering process of alkali-metal (left) and non-alkali metals (right) between two Fermi surfaces (FSs) in adjacent Brillouin zones (BZs) is shown in the upper panel, respectively. The momentum gap described by ${{\boldsymbol{q}}}_{{gap}}$, and ${{\boldsymbol{q}}}_{{ph}}$ is the scattering phonon momentum. In the bottom panel, there is a comparison of band structures as well as the potential optical transitions induced by N (red) and U (blue) electron scattering processes for alkali metals (left panel) and non-alkali metals (right panel), respectively. The green dashed lines correspond to the positions of the Brillouin zone. **b** The (${{\gamma }_{{ep}}}^{N},{{\gamma }_{{ep}}}^{U}$) resolved optical loss phase diagram among versatile plasmonic metals in terms of the quantitatively calculated U and N scattering-enabled e-p scattering rate at room temperature (*T* = 298 K). Here, the size of circles represents the magnitude of the total calculated damping rate

We first of all systematically evaluate the intrinsic optical losses of plasmonic metals out of the interband absorption windows, by quantifying the scattering rate γ from momentum-gap-resolved intraband optical transitions nearby the adjacent Fermi surfaces (Fig. 1). Here, minor loss components at room temperature (RT), stemming from interband optical transitions and surface effects, are neglected for simplicity in the entire analytic calculations. More specifically, the RT optical loss term *γ*, dominated by electron-phonon (e-p) scattering, is composed of normal (N) scattering within the 1st Brillouin zone (BZ) and umklapp (U) scattering across adjacent BZs, where processes from higher-order BZs are not included because of extremely low scattering rates (see more details in Supporting Information S.I.1). Analytic calculations on γ real that, alkali metals, due to the considerable “momentum gap” (note as ***q***_gap_ in Fig. 1a) between Fermi surfaces in adjacent BZs, require large-momentum phonons for momentum compensation, thereby suppressing the U-scattering rate (see Fig. 1a). While the N scattering rate ${\gamma }_{{ep}}^{N}$ for intraband transition is analytically proportional to the density of state of conduction electrons with ${\gamma }_{{ep}}^{N}\, \sim \,{n}^{3}$, where *n* is the free electron density (Supporting Information S.I.1). Global comparisons on the calculated optical losses of all potential elementary plasmonic metals are depicted in the two-dimensional phase diagram coordinated by ${\gamma }_{{ep}}^{N}$ and ${\gamma }_{{ep}}^{U}$. As clearly unraveled in Fig. 1b, the simultaneous suppression of U and N scatterings figures out potassium approachable to the lossless metal limit around the left bottom corner in Fig. 1b, overwhelming conventional plasmonic metals in zero-gap (***q***_gap_ = 0) scattering picture.

### Experimental validation

To experimentally validate the intrinsically low optical damping of potassium, it is essential to fabricate the metal films with high crystallinity, atomically smooth surfaces, and minimal oxidation. This requirement poses a substantial challenge, as potassium exhibits extreme chemical reactivity—even higher than that of sodium^27^. As a result, potassium films prepared by UHV deposition^16^ or embossing approaches^28^ often suffer from surface oxidation and limited crystallinity, leading to pronounced structural inhomogeneity. Moreover, the previous thermo-assisted spin-coating method that works well for sodium is not yet applicable for potassium, as it operates far from the conditions required for near-crystalline solidification^21^ (see more details in Supporting Information S.Ⅱ.1). These constraints have so far prevented reliable access to high-quality potassium films suitable for exploring their intrinsic plasmonic advances.

**Fig. 2:**
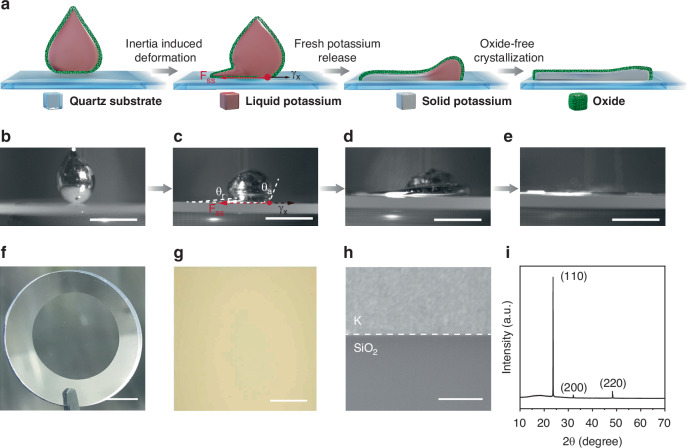
The SOC fabrication process of the high-quality potassium metal film. **a** Schematic of the SOC process and (**b**–**e**) are the optical images of the corresponding procedure taken by a high-speed camera (Scale bar: 5 mm). **f** The large-scale optical photograph of the prepared potassium film (Scale bar: 1 cm). **g** The partial magnification of the light microscope image (Scale bar: 300 μm). **h** FIB cross-section image of the K/quartz film. The dashed line clearly outlines the interface of the upper K film and the underlying quartz substrate, showing a flat morphology without evidence of an oxide layer (Scale bar: 1 μm). **i** XRD test result of prepared K film

Here, by decoupling the oxidation and solidification processes via ultrafast dicing of an oxide-encapsulated hot liquid droplet of potassium metal, we have developed the ultrafast slipping-assisted oxide-free crystallization (SOC) process to avoid oxidation and form a high-quality potassium surface. As schematically illustrated in Fig. 2a, after pre-removal of the native oxide and impurity shell using a vertically aligned quartz tube (Supporting Information, S.II.2) inside an inert-gas glovebox, the fresh potassium droplet undergoes free fall and adheres to a rapidly moving quartz substrate. Due to the inherent inertia and transformable geometry, the liquid droplet can not entirely follow the fast-moving substrate (see detailed kinetic discussion in Supplementary Information, Note S.II.3), and experiences a shear-stress-induced deformation on the fast-moving substrate (left panel in Fig. 2a), where the upper fluidic and lower static components of the droplet undergo quite different velocities. This finally triggers the droplet splitting, interfacial renewal and lateral spreading of fresh liquid potassium along the substrate (middle panel in Fig. 2a). As a result, solidification occurs within a confined, oxide-isolated interfacial region, producing a continuous and high-quality metal film (see high-speed optical images in Fig. 2b–e and Supplementary Movie S1).

The morphological and structural characteristics of SOC-prepared potassium films are summarized in Fig. 2f–i. Figure 2f shows a wafer-scale optical image of the ultra-smooth film, with a representative magnified micrograph in Fig. 2g. No detectable impurities or obvious defects are observed over areas exceeding 1 mm^2^ (see dark-field image in Fig. S16 for more details), demonstrating excellent lateral uniformity and scalability. This level of continuity and smoothness makes SOC-grown films suitable for both fundamental optical characterization and device fabrication involving highly reactive metals. The K/SiO_2_ interface is examined by cross-sectional SEM after FIB preparation (see Methods), as shown in Fig. 2h. The interface is ultra-flat and free of micro-bubbles or wrinkles, enabled by controlled interfacial wettability during rapid sliding-assisted solidification. XRD measurements (Supporting Information, Section S.II.5) reveal a dominant (110) diffraction peak, indicative of high crystallinity. The film quality significantly exceeds that of thermally prepared alkali-metal films under comparable non-vacuum conditions (Fig. S14). These results demonstrate that SOC enables non-vacuum fabrication of near-oxide-free, centimeter-scale potassium films with excellent smoothness and crystallinity, providing a robust platform for high-activity metal photonics and device integration.

**Fig. 3:**
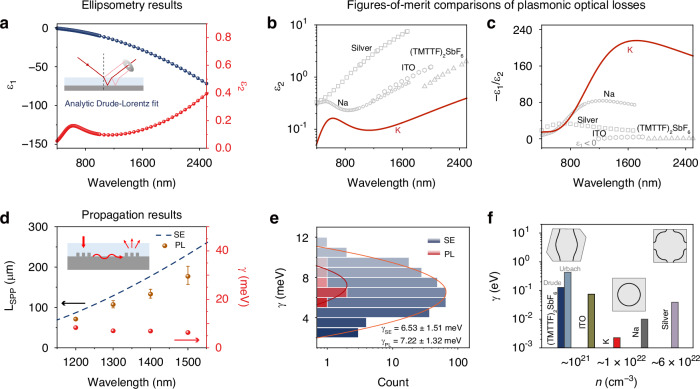
Optical losses of potassium characterized by far-field spectroscopy. **a** Real and imaginary parts of the dielectric functions (*ε* = *ε*_1_ + *iε*_2_) of the potassium film measured with a spectroscopic ellipsometer. The inset illustration shows the schematic diagram of the measurement method. **b**, **c** *ε*_2_ and -*ε*_1_/*iε*_2_ comparison with reported low-loss plasmonic metals (Ag, Na, ITO, and (TMTTF)_2_SbF_6_), respectively. **d** The propagation length and the *γ* retrieved from the propagation length method. The inset illustration shows the measured propagation length at different wavelengths of near-infrared on the potassium–quartz interface. The dotted line is the calculated propagation length based on the dielectric functions of potassium film measured with a spectroscopic ellipsometer. **e** The statistical data of *γ* retrieved from both SE and PL spectroscopic data. **f** Comparisons of experimentally reported milestone *γ* values of mainstream state-of-the-art plasmonic materials in terms of the free-carrier density and Fermi surface profiles

To quantify the intrinsic optical losses of the SOC-prepared potassium films, the dielectric functions were measured at ~ 300 points across a 5 mm × 5 mm area over 300–2500 nm using a commercial SE setup (RC2, J.A. Woollam Corporation). The results are well described by a standard Drude-Lorentz model without additional nonphysical parameters, expressed as the sum of intra- and interband contributions^29^:

1 $$\varepsilon (\omega )={\varepsilon }_{\infty }-\frac{{\omega }_{p}^{2}}{{\omega }^{2}+i\omega {\gamma }_{Drude}}+\frac{f{\omega }_{p}^{2}}{{\omega }_{1}^{2}-{\omega }^{2}-i\omega {\gamma }_{inter}}$$

where ${\varepsilon }_{\infty }$ is the background permittivity, ${\omega }_{p}$ is the bulk plasma frequency of potassium, ${\gamma }_{{Drude}}$ is Drude (momentum) damping rate of free carriers, $f$, ${\omega }_{1}$ and ${\gamma }_{{inter}}$ are the Lorentz amplitude, resonant frequency of the interband transition and interband damping rate, respectively. Figure 3a shows a typical fitted dielectric function (Detailed fitting parameters and the dielectric function fitting data are provided in the Supporting Information, Note S.Ⅰ.4). The weak absorption peak (~600 nm) corresponds to the well-known s-to-p interband transition of K (110). In the near infrared, the measured *ε*_2_ of K prepared by SOC is ~ 1/100 of Ag^30^ as well as ~ 1/10 of Na^21^ and transparent conductors such as ITO and (TMTTF)_2_SbF_6_^19^ (Fig. 3b). Figure 3c further compares the figure of merit (FOM, defined by −*ε*_1_*/ε*_2_^31^), demonstrating that SOC-prepared potassium exhibits state-of-the-art optical performance in the near-infrared, surpassing previously reported low-loss metals by about one to two orders of magnitude.

In addition to the low optical loss measured by SE, the damping rate γ can be independently extracted from the propagation length (PL) of surface plasmon polaritons (SPPs) along semi-infinite potassium-based plasmonic waveguides, providing independent confirmation of the low-loss nature of SOC-prepared K (Supporting Information, Note S.II.9). The fabrication of these plasmonic waveguides is detailed in Supporting Information, Note S.II.8. SPP intensity decay was measured at various propagation distances, and the corresponding propagation lengths at different excitation wavelengths were obtained by fitting the decay curves (Fig. S22). Figure 3d presents the experimental PL results (${L}_{{SPP}}$), with the extracted loss term *γ* shown on the right axis (the magnified plot of the loss term *γ* for the right axis is shown in Fig. S21). The lowest *γ* obtained from PL measurements reaches ~ 6.3 meV at 1500 nm. Figure 3e compares measured *γ* extracted from PL and SE measurements, showing a consistent wavelength-dependent trend—lower damping at longer wavelengths—consistent with previous reports^21^, as well as the minimal record low damping rate of 2.27 meV. The slight increase in *γ* observed in PL measurements is likely due to extra defects or impurities introduced during waveguide fabrication. Based on these independent measurements, we compare the intrinsic optical losses of SOC-prepared K with the state-of-the-art low-loss plasmonic candidates ranging from single-crystalline Ag^32^, sodium^21^, semiconductors ITO^33^ to hypergap transparent conductors^19^, as shown in Fig. 3f. Potassium exhibits optical damping rates that are ~1–2 orders of magnitude lower than these previously reported candidates, consistent with its reduced free-carrier density and near-parabolic Fermi surface with considerable momentum gap between adjacent BZs, which minimizes both U and N scattering rate as predicted in Fig. 1.

**Fig. 4:**
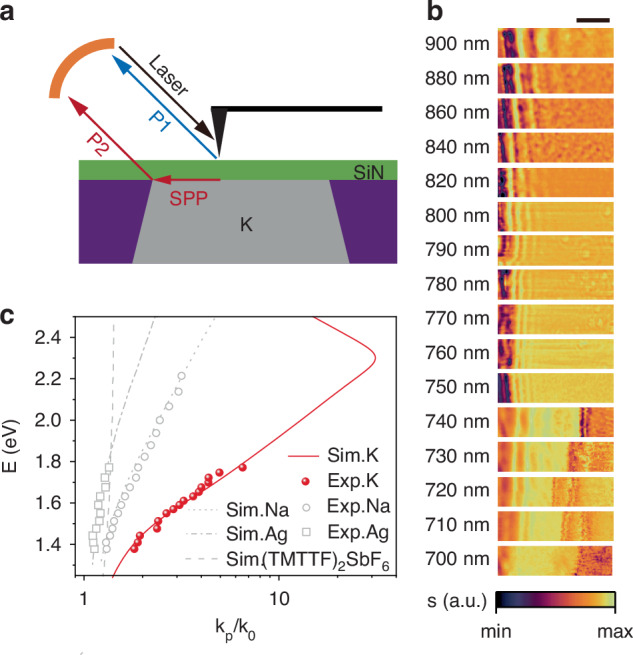
Low-loss initiated deep optical confinement of potassium by near-field spectroscopy. **a** Illustration of s-SNOM experimental setup to characterize K-SiN-Air SPP device. **b** s-SNOM imaging data of incident light range from 700–900 nm. The scale bar for 750–900 nm is 1 μm, for 700–740 nm is 0.5 μm. **c** Optical confinement comparison of SPP modes excited in the metal-SiN-Air device for K, Na, Ag and the bulk mode for (TMTTF)_2_SbF_6_, both in theoretical (line) and experimental (dotted) results

To investigate to what extent the remarkably reduced optical losses can enable strong plasmonic confinement in the experiment, near-field optical measurements were performed. We developed an ultrathin-film encapsulation technique for K–SiN–air devices under non-vacuum conditions (Supporting Information, Note S.II.13), which allows scattering-type scanning near-field optical microscopy (s-SNOM)^34^ to probe the dispersion of excited SPP modes (Fig. 4a). s-SNOM images were collected at incident wavelengths of 700–900 nm (Fig. 4b), and near-field fringe profiles were extracted (Fig. S23a), with corresponding Fourier transforms shown in Fig. S23b. The SPP wavevector is related to the fringe spacing *ρ* by

2 $$\frac{{k}_{p}}{{k}_{0}}=\frac{2\pi /\rho }{{k}_{0}}+cos\alpha$$

where $\alpha =36^\circ$ is the incident angle of the laser beam relative to the sample plane. The extracted (*k*_*p*_*/k*_0_, E) points (Fig. 4c) match well with calculated SPP dispersion (Supporting Information, Note S.I.5). The effective index *k*_*p*_*/k*_0_ for potassium exceeds 10, nearly one order of magnitude larger than low-loss silver and the emerging transparent conductor (TMTTF)_2_SbF_6_. The latter exhibits metallic behavior only for *λ* > 1900 nm, and its apparent field confinement at shorter wavelengths is due to the high bulk refractive index rather than intrinsic SPP compression. The experimental achievement of the deep-subwavelength confinement of potassium that beats mainstream state-of-the-art plasmonic materials is primarily stemmed from the more favorable permittivity (negative but smaller magnitude of *ε*_1_) as well as ultralow loss *ε*_2_ (see more detailed discussions in Supporting Information S.I.7 and Figure S6). Further optimizations are promising once extra low-loss materials and/or structural designs are introduced. For instance, by vertically integrating the ultrathin encapsulated potassium with low-loss optical metastructures such as multilayer or hyperbolic metamaterials^35^, the optical field can be further compressed. Searching for potassium-like new plasmonic materials, with global loss reduction by combining both the momentum-gap-based scattering model and hypergap idea^19^, or with reduced Landau damping—dominating at extremely large wavevectors^27^, deep nanoscale field concentration with ultralow plasmonic losses can be ultimately expected. These results highlight potassium’s exceptionally low intrinsic optical loss and its potential for extreme SPP confinement in room-temperature devices.

## Discussion

In this work, we have demonstrated, both theoretically and experimentally, the potential of potassium as a low-loss plasmonic metal enabled by the SOC fabrication process. The suppression of U-scattering-related absorption and the achievement of oxide-free crystallization result in potassium films with an exceptionally low imaginary part of the permittivity (~0.1) across the visible and near-infrared range, corresponding to a damping rate of 2.27 meV, nearly 1–2 orders of magnitude lower than that of sodium, noble metals and other transparent conductors reported thus far. These properties allow K-based SPP devices to achieve extremely deep-subwavelength optical confinement, with a compression factor approaching 10 even in unpatterned structures. Overall, our findings establish a practical pathway toward the intrinsic low-loss limit of plasmonic metals and provide new opportunities for deep-subwavelength photonics.

## Methods

### FIB fabrication

We use a gallium-ion-equipped double-beam electron microscope (Scios DualBeam from FEI, 30 kV) to cut a cross-section of the potassium film. The active potassium film sample was transferred to the electron microscope chamber with a vacuum box to prevent oxidation by air (Quick Loader IGST SDB from FEI).

## Supplementary information

Supplementary_Materials_for_readers

Movie S1

## Data Availability

The data that support the findings of this study are available from the corresponding authors upon reasonable request.
