# Supplementary material for: Approaching the low optical loss limit of plasmonics using potassium
Source: Light Sci Appl. 2026 Jul 21;15:326. doi: 10.1038/s41377-026-02400-8 (PMC13389207; doi:10.1038/s41377-026-02400-8)
Supplement: Supplementary file 1 — Supplementary_Materials_for_readers [file 41377_2026_2400_MOESM1_ESM.docx]

**Supplementary Information for**

**Approaching the Low Optical Loss Limit of Plasmonics using Potassium**

Yi Zhang^1,2^†, Yuhan Yang^1^†, Jie Liang^1^†, Shizhuang Wang^1^†, Yuhong Shi^1^, PengCheng Yao^1^, Hanyu Fu^1^, Jacob B. Khurgin^3^*, Fengrui Hu^1^, Jia Zhu^1^, Shining Zhu^1^, Lin Zhou^1^*

*Corresponding author: [linzhou@nju.edu.cn](mailto:linzhou@nju.edu.cn); jakek@jhu.edu

**Contents**

S. Ⅰ The momentum-gap based scattering model

S. Ⅰ. 1 Calculation of intraband transition induced by three kinds of scattering

S. Ⅰ. 2 First ionization energy of alkali metals

S. Ⅰ. 3 DFT calculations of binding energy

S. Ⅰ. 4 The fitting parameters obtained for the potassium film

S. Ⅰ. 5 Calculation of dispersion curve of multilayer Air/SiN/Metal devices

S. Ⅰ. 6 Evaluation on oxidation layers of K and Na

S. I. 7 High confinement analysis

S. Ⅱ Experimental characterizations for the prepared potassium

S. Ⅱ. 1 Potassium film fabricated by the thermal-assisted spin-coating method

S. Ⅱ. 2 The oxide removal process

S. Ⅱ. 3 The crystallization process

S. Ⅱ. 4 Preparation process of ultra-smooth potassium samples for XRD tests

S. Ⅱ. 5 Crystalline nature comparison of potassium and sodium film fabricated by different methods

S. Ⅱ. 6 Analysis of the optical image of the prepared potassium film

S. II. 7 Dielectric function comparison of different materials

S. Ⅱ. 8 The fabrication process of the potassium-based plasmonic waveguide

S. Ⅱ. 9 Structure of semi-infinite SPP waveguide for measuring propagation length

S. Ⅱ. 10 The γ retrieved from propagation length and compared with sodium from previous literature

S. Ⅱ. 11 Propagation measurements for potassium film with exponential curves fitted to the data

S. Ⅱ. 12 Optical loss extracted from propagation length through plasmonic waveguide device

S. Ⅱ. 13 Fabrication of metal/SiN/Air SPP device for s-SNOMs test

S. Ⅱ. 14 Real-space fringe profiles and corresponding FT profiles of the s-SNOM imaging data

S. Ⅱ. 15 Time-dependent measurements for stability characterization

S. Ⅲ Supplementary movies

S. Ⅲ. 1 Movie S1. Side-view of the stage of crystallization

**S. Ⅰ The momentum-gap based scattering model**

**S. Ⅰ. 1 The calculation of intraband loss induced by three kinds of scattering**

The plasmonic damping in optical range are well known as the sum of three damping rates of $\gamma_{ep}$, $\gamma_{ee}$ and $\gamma_{es}$with respect to the electron-phonon (*e-p*), electron-electron (*e-e*), and electron-surface scatterings (*e-s*), respectively, reading as $\gamma_{intra}=\gamma_{ep}+\gamma_{ee}+\gamma_{es}.$

We start with the electron-phonon damping term* *$\gamma_{ep}$ which can be decomposed into two sub-terms (${\gamma_{ep}}^{N},{\gamma_{ep}}^{U})$ independently enabled by normal (N) and umklapp (U) scatterings^1–3^, respectively, $\gamma_{ep}={\gamma_{ep}}^{N}+{\gamma_{ep}}^{U}$. At room temperature, the ${\gamma_{ep}}^{N}$ term can be evaluated based on the 1^st^ and 2^nd^ plane-wave model in terms of pseudopotential theory^4,5^. The expression for the normal e-p scattering at room temperature is given by.

${v_{ep}}^{N}=\frac{\pi{E_{d}}^{2}n_{F}}{2\hbar\rho{v_{s}}^{2}}k_{B}T$ (S1)

where $E_{d}$ is deformation potential, $n_{F}$ is density of states at $E_{F}$ (where $E_{F}={\hbar^{2}{(3\pi^{2}n)}^{3/2}}/{2m}$ is Fermi energy, where *n* and *m* are density and mass of electron, respectively), $\rho$ is the mass density of materials and $v_{s}$ is velocity of sound in the material. For free electron model, the deformation potential is equal to $-2{E_{F}}/3$. Substituting the result into Eq.(S1), one can obtain the expression of ${\gamma_{ep}}^{N}={(3\pi^{7}\hbar^{3}n^{3}n_{F}k_{B}T)}/{2m\rho{v_{s}}^{2}}$.

However, for the Umklapp e-p scattering, the rate of e-p scattering can be written as^6^

${\gamma_{ep}}^{U}=\alpha{\gamma_{ep}}^{N}$ (S2)

where $\alpha=\frac{v_{1}}{v_{2}}\left( \frac{E_{d,t}}{E_{d,l}} \right)^{2}\left( \frac{s_{l}}{s_{t}} \right)^{4}I(x_{0})$, $v_{1}$ and $v_{2}$ are the electron velocities on the two Fermi surfaces located in two adjacent Brillouin zone, respectively, and the $E_{d,t}$ and $E_{d,l}$ are longitudinal and transverse components of the deformation potential responsible for scattering by the longitudinal and transverse phonons with velocities $s_{t}$ and $s_{l}$, and $I\left( x_{0} \right)=\int_{x_{0}}^{\infty} x^{2}\Phi(x)dx$, where $x_{0}={\hbar q_{min}s}/{k_{B}T}$ (here $s$ is the average velocity of sound in the material and $q_{min}$ is the smallest momentum gap (also noted as q_gap_ in Fig. 1a) between the two Fermi surfaces in two adjacent Brillouin zones) and $\Phi\left( x \right)=\frac{4}{(e^{x}-1)(e^{-x}+1)}$ is occupation factor at fermi level. The method of calculation of $E_{d,t(l)}$ can be found in ref.^7^ and ref^8^.


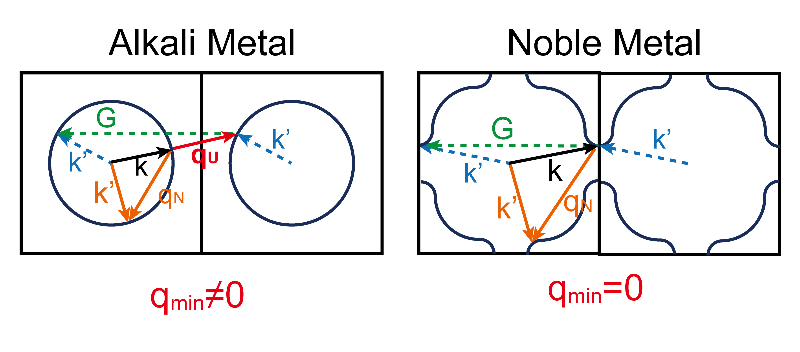


**Figure S1.** A comparison of electron–phonon scattering induced by N (red) and U (blue) electron scattering processes for alkali metal (left panel) and noble metals (right panel), respectively (here *k* and *k’* are initial and final momentum of electron, $q_{N}$ and $q_{U}$ are phonon involved in N and U scattering, respectively, *G* is reciprocal lattice vector.) and $q_{min}$ is the minimum gap between Fermi surfaces in two adjacent Brillouin zones.

Although the e-e scattering is negligible at room temperature, a simple theoretical treatment is included here. Several works^2,9^ made evident that the umklapp scattering of electrons is major source of the optical loss of metals at low temperature. Therefore, that means the umklapp scattering is the main mechanism of optical loss in metals induced by e-e scattering. The e-e scattering rate can be written as:

$\gamma_{ee}=\frac{\pi^{3}\Gamma\Delta}{12\epsilon_{F}}\left[ \left( k_{B}T \right)^{2}+\left( \frac{\hbar\omega}{2\pi} \right)^{2} \right]$, (S3)

where $\Gamma$, $\Delta$ , $\epsilon_{F}$ and $k_{B}$are the average scattering probability, fractional umklapp scattering, Fermi energy and Boltzmann constant, respectively. The methods of calculation of $\Gamma$ and $\Delta$ are given in Ref. [5].

Although not discussed in the main text, the low-loss advantage of potassium remains even with the inclusion of electron-surface scattering (e-s scattering) effects. As for e-s scattering, it can be calculated by the formula^10^

$\gamma_{es}=\frac{3}{8}\frac{v_{F}}{\delta}$ (S4)

where $v_{F}={\hbar\sqrt[3]{3\pi^{2}n}}/m$ is Fermi velocity, where $m$ and $n$ are mass and density of electron respectively, $\delta$ is the skin depth. The loss due to the e-s scattering is, in fact, the Landau damping or Kreibig damping. Due to combined lower Fermi velocity and large skin depth ($\delta=\sqrt{2/{\omega\mu\sigma}}$, where $\omega$ is the frequency of incident light,$\mu$ is permeability and $\sigma$ is conductivity) compared to other metals, alkali metals exhibit extremely low e-s scattering rate. As an example, based on the corresponding data of $v_{F}$ and $\delta$, the electron-surface scattering rate of potassium is about $0.81\times{10}^{13}s^{-1}$, while that of silver and gold are $4.17\times{10}^{13}s^{-1}$ and $5.25\times{10}^{13}s^{-1}$, respectively.

**S. Ⅰ. 2 First ionization energy of alkali metals**


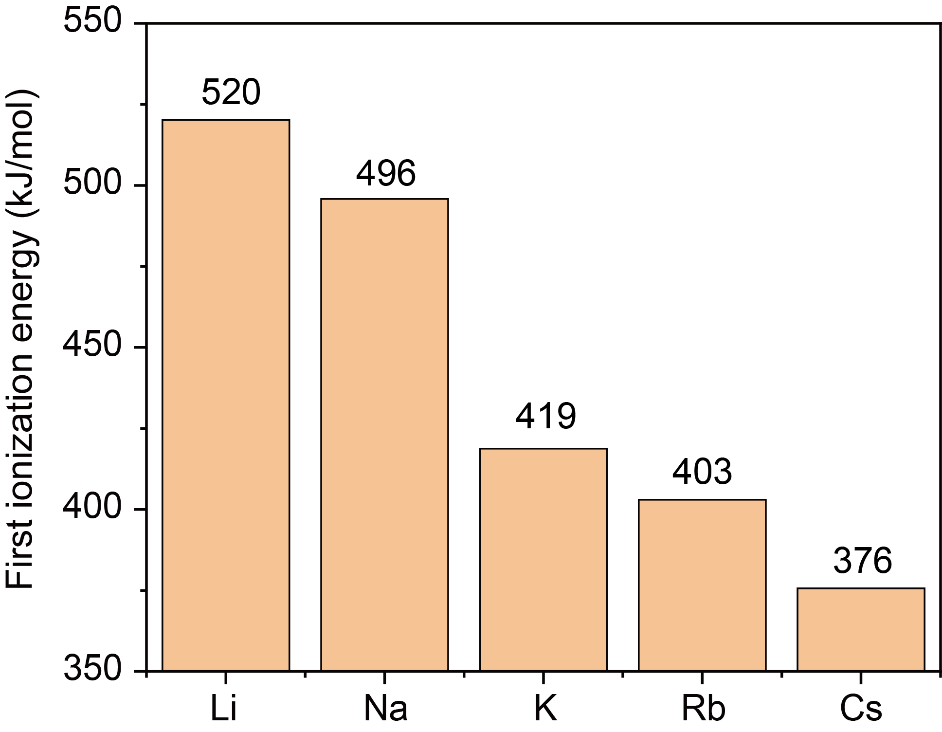


**Figure S2.** First ionization energy of alkali metals. The first ionization energy of K relative to Na is reduced by 15%, which is higher than that of other metals in the same group (~ 5%).

**S.** **Ⅰ. 3 DFT calculations of binding energy**

In order to figure out the mechanism of quartz peeling process, we made DFT calculations of the binding energy between K_2_O/K pair and K_2_O/SiO_2_ using the CASTEP code integrated into the Materials Studio software. Ultrasoft pseudopotential as implemented in the CASTEP code was applied. PBE form of generalized-gradient approximation (GGA) was adopted. The K/K_2_O pair was confined in a 5.32*7.52*43.34 $Å^{3}$ periodic box, with the K (1,1,0) surface and K_2_O (1,1,0) surface initially exposed. The K/SiO_2_ pair was confined in a 5.05*5.05*34.94 $Å^{3}$ periodic box, with the SiO_2_ (0,0,1) surface and K_2_O (1,1,0) surface initially exposed. K, K_2_O, and SiO_2_ alone, as well as the K/K_2_O pair and K/SiO_2_ pair all underwent geometry optimization, respectively, without specific lattice symmetry restrictions to simulate the amorphous state of each material. The geometry optimization was done by means of the Broyden-Fletcher-Goldfarb-Shanno (BFGS) algorithm, allowing all atomic dimensions to vary to reach the minimum energy state. The binding energy of materials A and B was calculated from the equation below:

$E_{binding}=E_{A}^{total}+E_{B}^{total}-E_{A/B}^{total}$ (S5)

The optimized superlattice structures and binding energies per unit area are shown in **Figure S3**. The result indicates that K/K_2_O pair has a lower binding energy than K/SiO_2_ pair, which enables the K_2_O attach to SiO_2_ substrate at initial point.


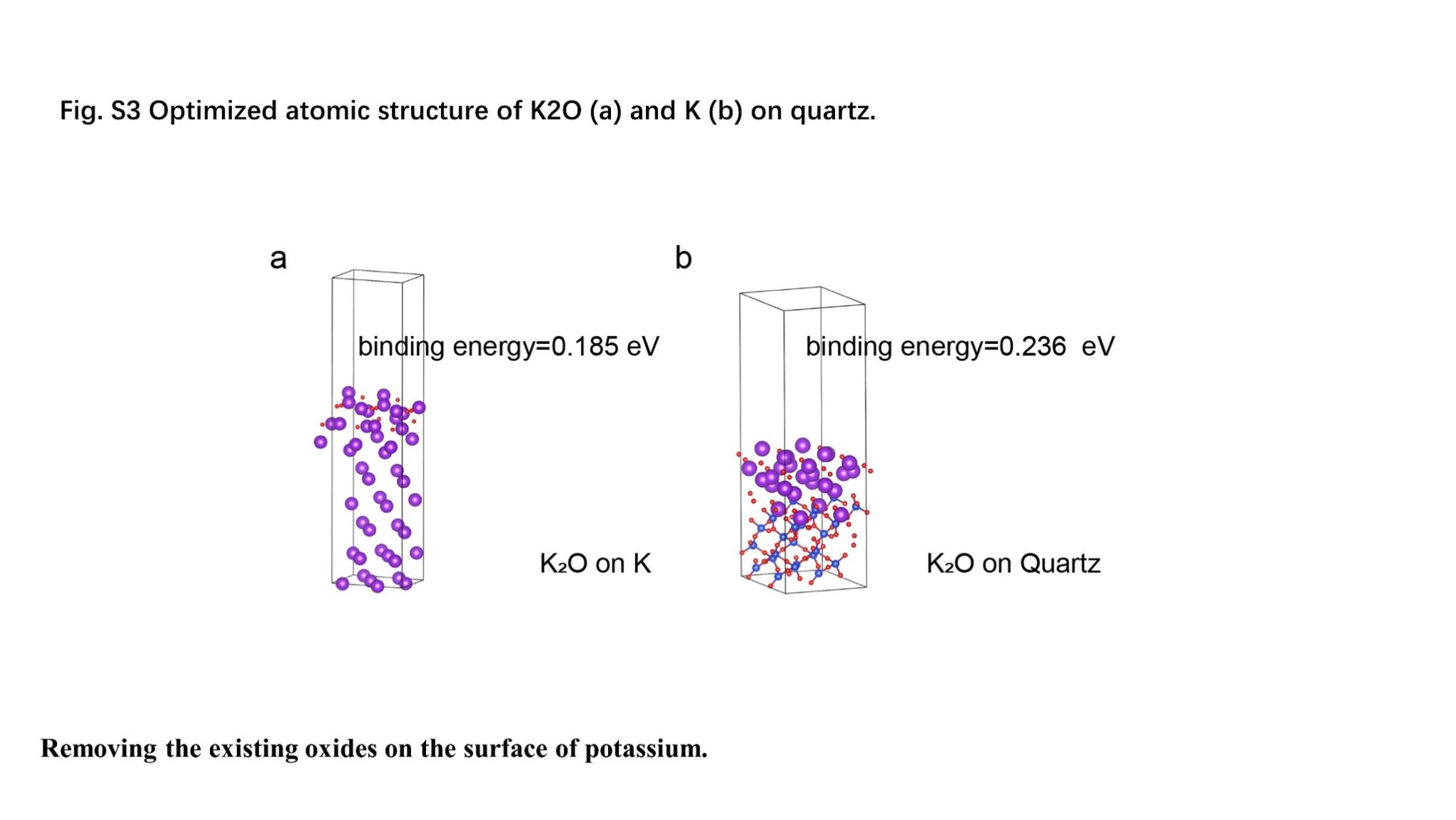


**Figure S3.** Optimized atomic structure of K_2_O (a) and K (b) on quartz. The binding energy between K_2_O and K ($E_{K_{2}O/K}$) is lower than that between SiO_2_ and K ($E_{K_{2}O/{SiO}_{2}}$).

**S. Ⅰ. 4 The fitting parameters obtained for the potassium film**

The fitting parameters obtained for the potassium film in Figure 3a are$\varepsilon_{\infty}$=1.171,$\omega_{p}^{2}$=17.991 eV^2^, γ_Drude_=0.00254 eV, *f*=0.151737, $\omega_{1}$=1.5763 eV, γ_inter_=2.143 eV., as shown in **Figure S4**. The relative dielectric function fitting data are provided in Supplementary file 1.


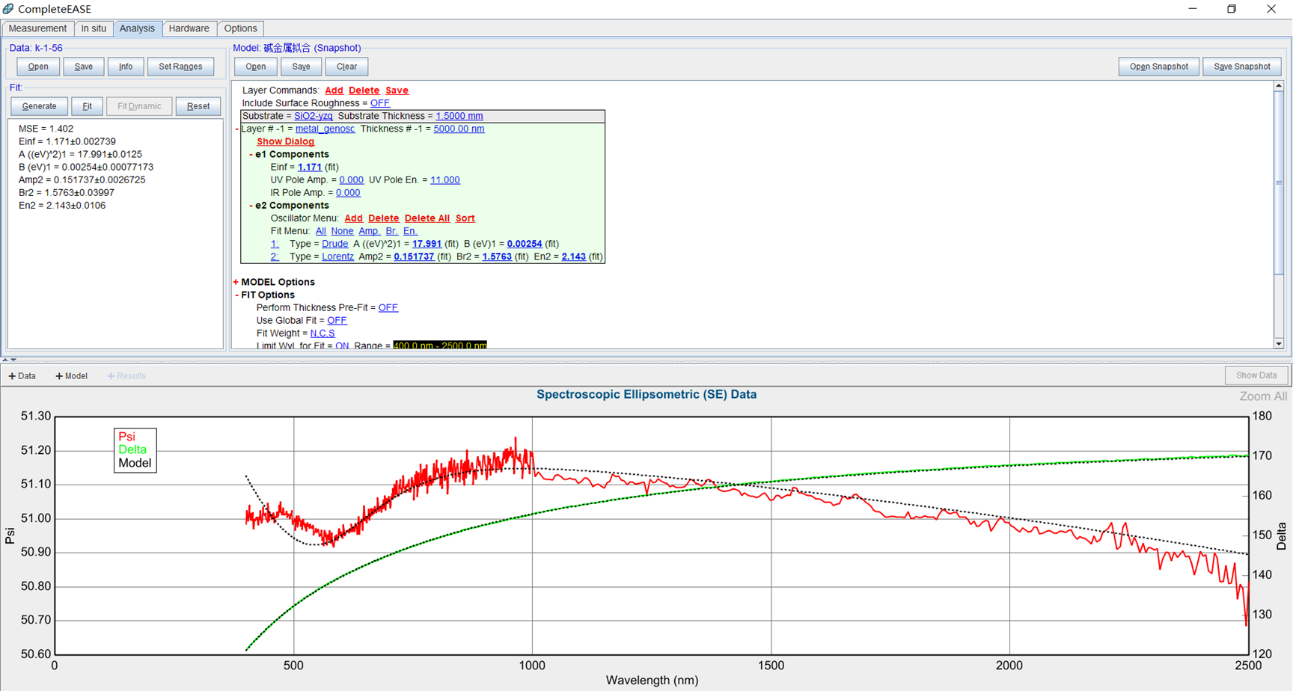


**Figure S4**. The ellipsometric fitting parameters of our best potassium metal.

**S. Ⅰ. 5 Calculation of dispersion curve of multilayer Air/SiN/Metal devices**

The dispersion curve of our multilayer K-SiN-Air devices in **Figure 4c** was obtained from analytical solution derived from Maxwell's equations. Considering the electric field right underneath the s-SNOM probe is perpendicular to the sample surface, only transverse magnetic (TM) waveguide modes are excited.

We utilized a multilayer planar waveguide structure to calculate the analytical solutions of the SPP modes excited in the Metal-SiN-Air devices. Due to the trace amounts residual oxygen at scattering edges observed in experiment, which tend to interact with the reactive metal (especially potassium) at the interface of metal and SiN, we consider an additional oxide layer ($\varepsilon_{2}$ = 2.25) between the SiN ($\varepsilon_{1}$ = 4.62) and metal layer as a correction layer. The structure is shown in **Figure S5**. The dispersion equation for the SPP modes excited within the multilayer structure is derived using Maxwell's equations and boundary conditions, as follows:

$e^{-2k_{2}b}=\frac{Y_{1}(\frac{k_{4}}{\varepsilon_{4}}+\frac{k_{2}}{\varepsilon_{2}})}{Y_{2}(\frac{k_{4}}{\varepsilon_{4}}-\frac{k_{2}}{\varepsilon_{2}})}$ (S6)

$Y_{1}=\frac{2k_{1}}{\varepsilon_{1}}\left( \frac{k_{3}}{\varepsilon_{3}}+\frac{k_{1}}{\varepsilon_{1}} \right)e^{2k_{1}a-k_{2}a}-(\frac{k_{2}}{\varepsilon_{2}}-\frac{k_{1}}{\varepsilon_{1}})e^{-k_{2}a}[\left( \frac{k_{3}}{\varepsilon_{3}}-\frac{k_{1}}{\varepsilon_{1}} \right)-\left( \frac{k_{3}}{\varepsilon_{3}}+\frac{k_{1}}{\varepsilon_{1}} \right)e^{2k_{1}a}]$(S7)

$Y_{2}=\frac{2k_{1}}{\varepsilon_{1}}\left( \frac{k_{3}}{\varepsilon_{3}}+\frac{k_{1}}{\varepsilon_{1}} \right)e^{2k_{1}a+k_{2}a}+(\frac{k_{2}}{\varepsilon_{2}}+\frac{k_{1}}{\varepsilon_{1}})e^{k_{2}a}[\left( \frac{k_{3}}{\varepsilon_{3}}-\frac{k_{1}}{\varepsilon_{1}} \right)-\left( \frac{k_{3}}{\varepsilon_{3}}+\frac{k_{1}}{\varepsilon_{1}} \right)e^{2k_{1}a}]$(S8)

$k_{1}=\sqrt{k_{p}^{2}-k_{0}^{2}\varepsilon_{1}}$ (S9)

$k_{2}=\sqrt{k_{p}^{2}-k_{0}^{2}\varepsilon_{2}}$ (S10)

$k_{3}=\sqrt{k_{p}^{2}-k_{0}^{2}\varepsilon_{3}}$ (S11)

$k_{4}=\sqrt{k_{p}^{2}-k_{0}^{2}\varepsilon_{4}}$ (S12)

Here, 𝜀 represents the dielectric constant of the respective layer, 𝑎 = 40 nm is the thickness of the SiN layer, set to 40 nm, and 𝑏 is the thickness of the oxide correction layer. For the K system, 𝑏 is set to 3 nm; for the Na system, it is set to 2 nm (Compared with K, Na exhibits lower chemical reactivity, thus a thinner layer is assumed); and for the Ag system, it is set to 0 nm. The thickness 𝑏 is proportional to the chemical reactivity of the three metals.


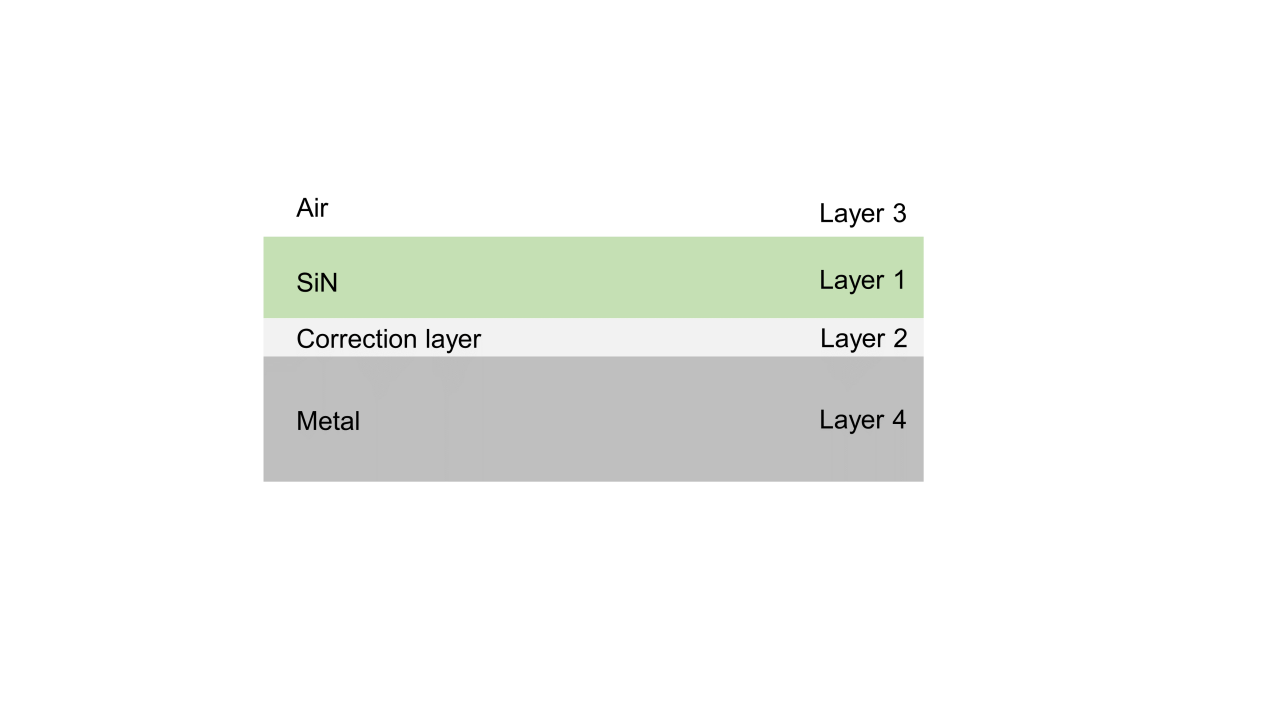


**Figure S5.** The illustration of the Air/SiN/Correction layer/Metal multilayer planar structure.

**S. Ⅰ. 6 Evaluation on oxidation layers in K and Na**

In the SOC process in which potassium undergoes an extremely short exposure to ultra-low oxygen concentrations (< 0.01 ppm O_2_), the thickness of the resulting oxide layer is primarily determined by the amount of oxygen consumed. We assume that the oxidation process is mass-transport limited by the scarcity of oxygen rather than the intrinsic reaction rate. Under these conditions, we assume the molar consumption of oxygen per unit area is comparable for both samples, leading exclusively to the formation of monoxides (Na_2_O and K_2_O) without progressing to peroxide or superoxide, which would require more oxygen and longer reaction time.

Consequently, the difference in film thickness is primarily dictated by the molar volume (V_m_) of the resulting oxide phases. Using standard densities (2.27 g cm^-^³ for Na_2_O and 2.35 g cm^-^³ for K_2_O), we calculated the V_m_ of Na_2_O to be approximately 27.3 cm³ mol^-1^, while that of K_2_O is 40.1 cm³ mol^-1^. This geometric expansion alone suggests the K-oxide film should be roughly 1.47 times thicker than the Na-oxide film for the same amount of consumed oxygen, consistent with the approximately 1.5-fold value reported in Supplementary S. I. 5*.*

**S. I. 7 Roles of plasmonic losses in achieving high optical confinement**

The high confinement of SPP in the near-field K/dielectric configuration is not only determined by ε_1_ but also highly dependent on ε_2_. The underlying mechanism is that, the high optical loss will cause bending of the dispersion curve backward, making high-k states (with high confinement) practically inaccessible.

To provide a qualitative physical picture, consider the simplest K/Air SPP case. The spatial confinement is defined by free-space wavelength over the SPP wavelength (λ_0_/λ_spp_), which can be derived from the SPP dispersion equation *k_spp_* = *k*_0_[(ε_1_+iε_2_) ε_d_/(ε_1_+iε_2_+ε_d_)]^1/2^. By applying the approximation condition of |ε_1_|>>ε_2_, the real and imaginary part of the SPP wavevector (*k_spp_* = *k*_1_ +i*k*_2_) is given by

(S13)

It is clear that, as ε_1_ approach -ε_d_, the spatial confinement (*k*_1_/*k*_0_) approaches infinity while the propagation loss ε_2_/*k*_0_ tends to infinity as well, validating that the predicted infinite field confinement becomes illusive in experiment as the existence of plasmonic loss (non-zero ε_2_).

More quantitatively, the loss-resolved dispersion curves for both K/Air and Ag/Air SPP configurations are calculated as shown in Figure S6c. One can clearly find that, the existence of optical losses results in the band bending and thus distinctly reduction of the achievable maximal value of k_spp_ (weaker confinement). Note that, even though the ε_1_ of potassium is closer to unity (with near-zero denominator in the upper equation), a higher plasmonic loss in K/Air geometry can even shrink the maximal k_spp_ distinctly smaller that that of the Ag/Air counterpart.


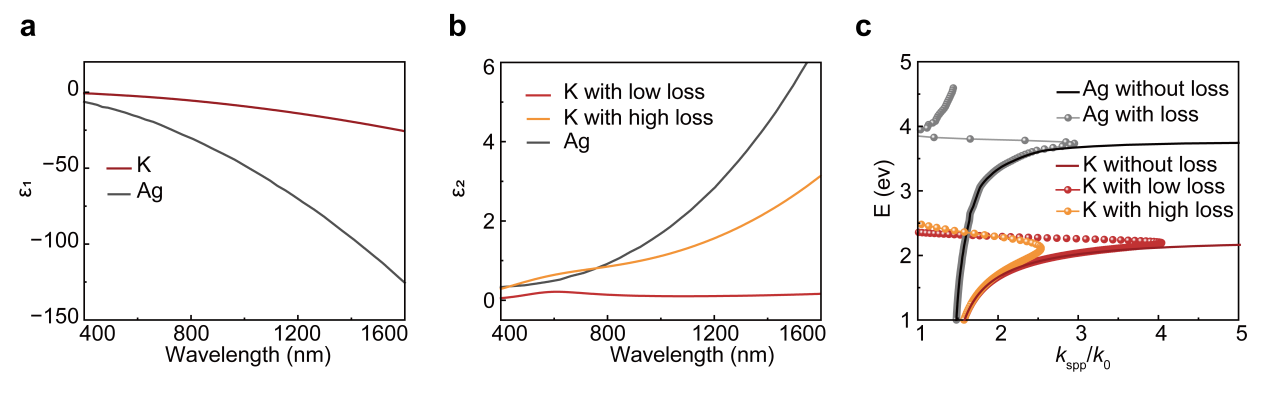


**Figure S6.** (a) Real part of the dielectric function Re(ε) of Ag and K. (b) Imaginary part Im(ε) of Ag and K. (c) Calculated SPP dispersion diagrams for three semi-infinite metal/dielectric configurations: real Ag with low loss (black dotted lines), real K with low loss (red dotted lines), K with high loss (yellow dotted lines). Two solid lines with for the lossless Ag and K alternatives are included as well.

**S. Ⅱ Experimental characterizations for the prepared potassium**

**S. Ⅱ. 1 Potassium film fabricated by the thermal-assisted spin-coating method:**

We have tried employing the conventional thermal-assisted spin-coating method^11^ to fabricate potassium films (**Figure S7a**). However, potassium’s greater reactivity with oxygen than sodium leads to the newly formed oxide nearly enveloping the target substrate’s surface during fabrication (**Figure S7b**). Additionally, optical microscopy uncovers randomly distributed impurities (**Figure S7c**). **Figure S8** presents the ellipsometry data of potassium films fabricated by the thermo-assisted spin-coating method, together with a comparison to sodium films prepared using the same approach. The imaginary parts of the dielectric functions do not reveal the theoretically predicted intrinsic low-loss advantage of potassium, indicating that the thermo-assisted spin-coating method is not suitable for accessing the intrinsic optical performance of potassium films.

We further compare the statistical values of γ for potassium and sodium films fabricated using the same SOC method, as shown in **Figure S9**. In this case, potassium consistently exhibits a lower damping rate than sodium, demonstrating its intrinsically lower optical loss when oxidation is effectively suppressed. These results highlight the advantage of the SOC method in realizing the intrinsic plasmonic performance of chemically active potassium.


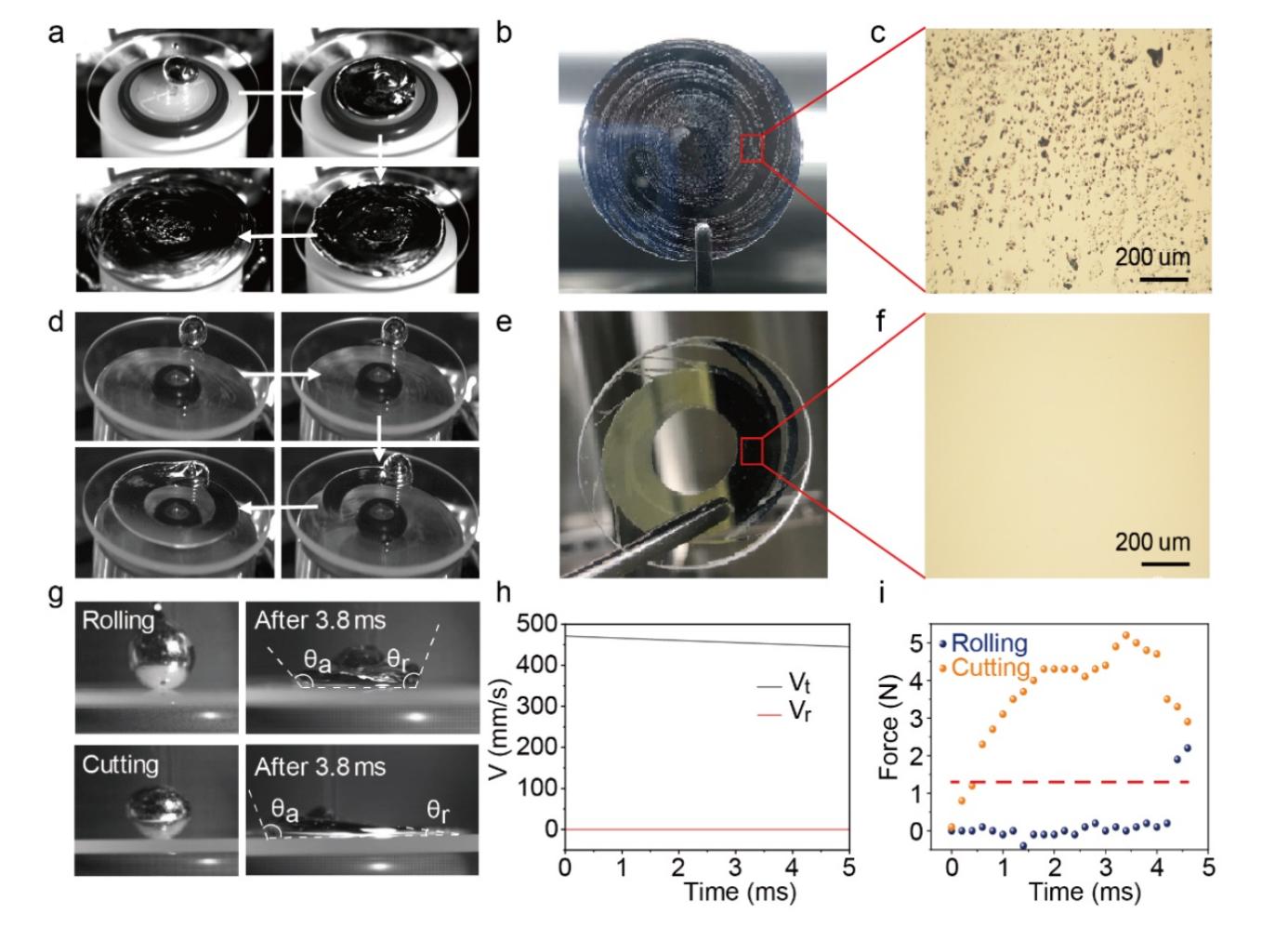


**Figure S7.** a) Experimental process of the thermal-assisted spin-coating^11^. b) Photograph of potassium film fabricated by thermal-assisted spin-coating method. c) Partial magnification of the light microscope image of the potassium film.


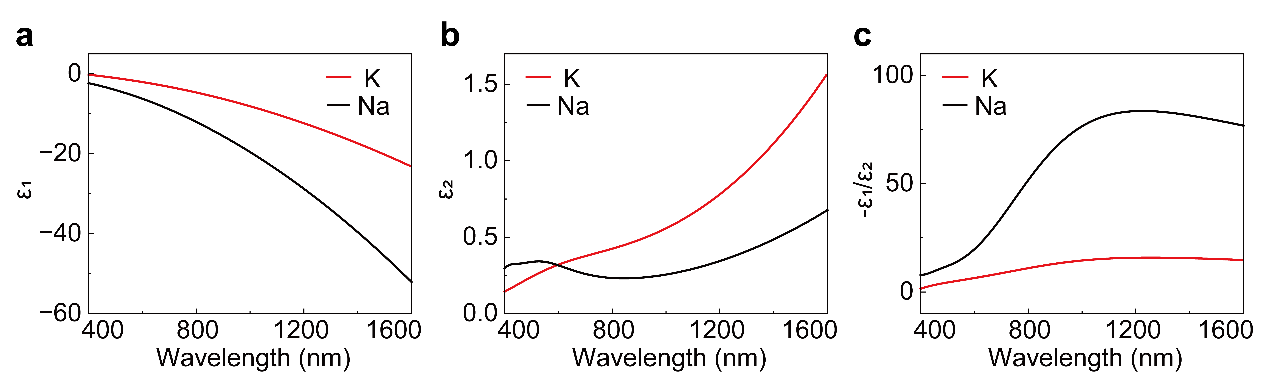


**Figure S8.** Comparison of the dielectric functions and figure of merit (-ε_1_/ε_2_) of potassium film and sodium film^11^ fabricated by thermal-assisted spin-coating method, measured with the spectroscopic ellipsometer.


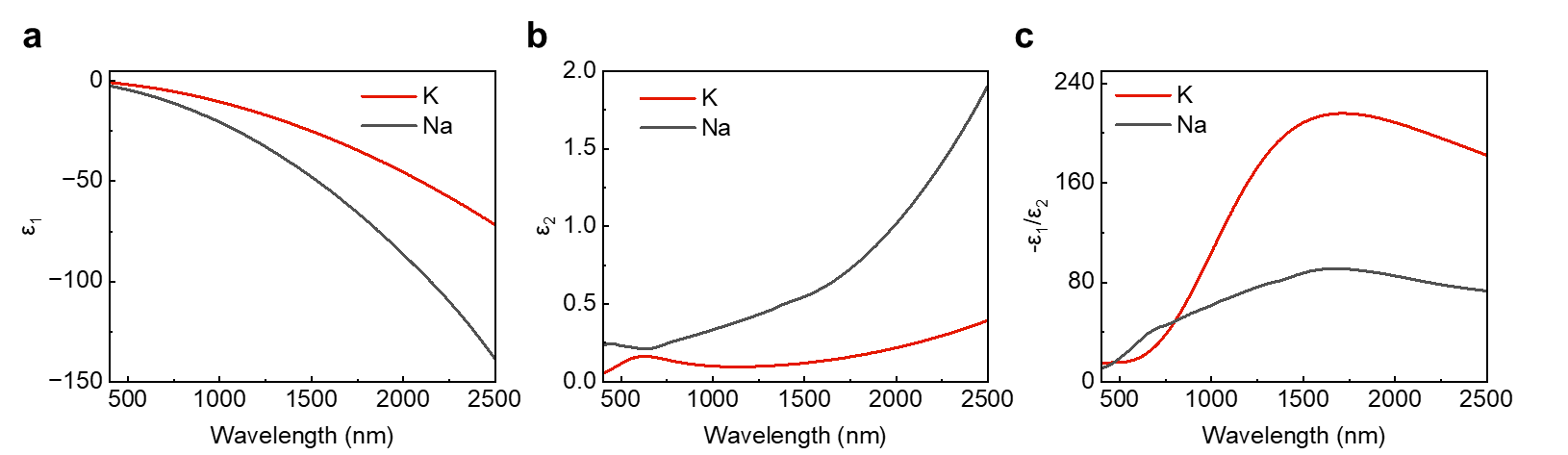


**Figure S9.** Comparison of the γ of potassium film and sodium film fabricated by SOC method, measured with the spectroscopic ellipsometer.

**S. Ⅱ. 2 The oxide removal process**

By heating the quartz tube to ~ 120 ℃ (above potassium's melting point of ~ 64 ℃), we maintained the internal potassium in a liquid state. Then, we squeezed the latex at tube’s head to keep the liquid K flow through the quartz tube. This process effectively removed the original oxide layer, leaving an oxide shell on the quartz tube's inner surface. Enlargement of **Figure S10a** shows the schematic and optical photograph of the process, with purified liquid on the topside of the tube and the remaining oxide trail on the underside. XRD analysis of the residual white layer on the quartz sidewall in **Figure S10b** confirmed it as K_2_O. Based on this process, the fresh potassium flow is available from the spray head of the heated quartz tube for further film preparation.


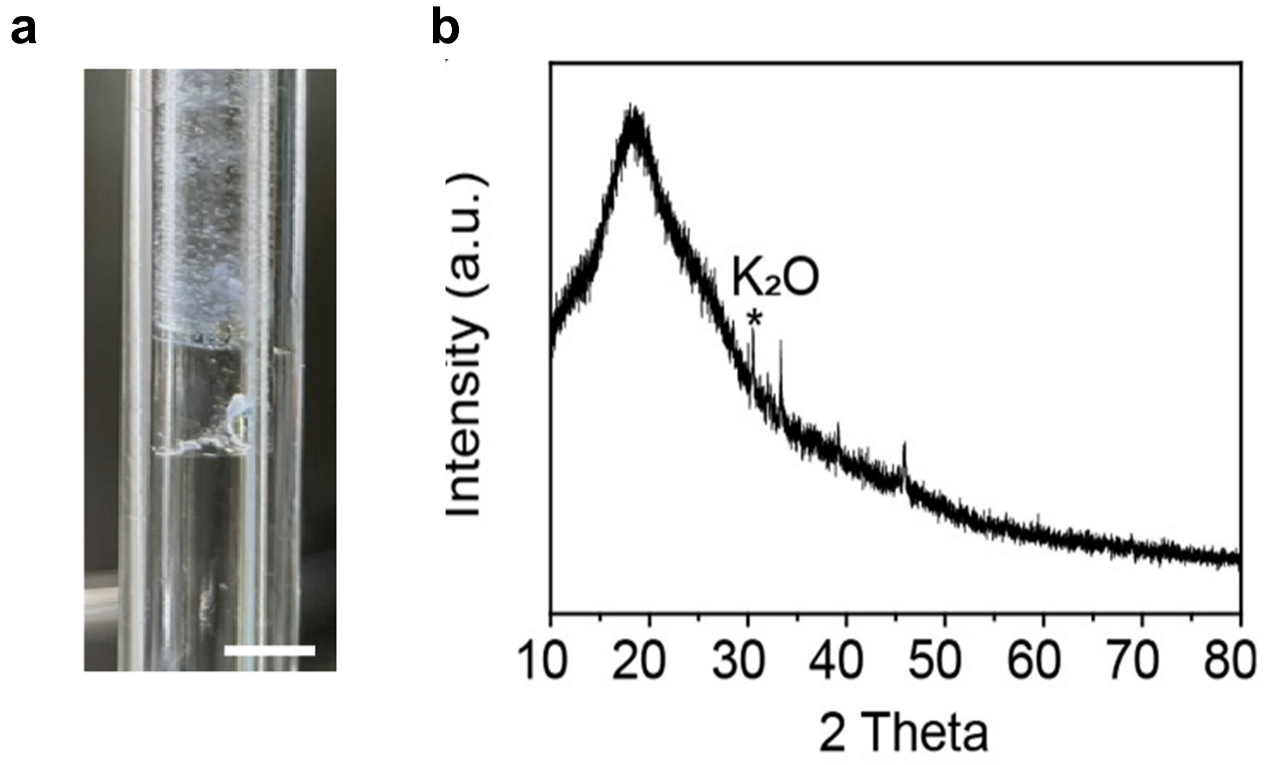


**Figure S10.** (a) The optical paragraph of oxide trail left on quartz tube. (b) XRD test result of the oxide shell on the trail after the liquid potassium flows through.

**S. Ⅱ. 3 Underlying mechanisms of the oxide-free crystallization process**

Once the fresh potassium is produced, the key is to form the high-crystal quality potassium before further oxidation. More intuitively, the oxide-free solidification of potassium on quartz is enabled by the simultaneous inertial and viscous response of the liquid potassium droplet to the rapidly moving quartz substrate. The mechanism can be described more accurately as follows:

During the droplet deposition process, the lower part of the droplet first contacts the quartz surface and is dragged by the moving substrate due to viscous coupling, while the upper part of the droplet cannot follow the motion immediately because of inertial effects. This velocity gradient induces shear deformation within the droplet, which promotes continuous spreading of liquid K and subsequent solidification in an oxide-isolated interfacial region, ultimately leading to the formation of a high-quality metal film.

As illustrated in **Figure 2c-e**, the droplet undergoes a free-fall and slips against a rapidly moving ultra-flat quartz substrate (act as the sharp blade).While it is still possible to form a thin potassium oxide during this period, the large tangential-velocity mismatch between potassium liquid (with a thin oxide) and quartz substrate induces huge lateral sheer stress (*F_SS_*) larger than the horizontal component of surface tension (*γ_x_*), allowing fresh liquid potassium to flow out and directly adhere to the substrate without oxygen exposure (**Figure 2c**). The potassium then solidifies along the path, forming a high-quality K film (**Figure 2d**) with an oxide-free interface at the substrate.

More specifically, when K droplet slides on an ultra-flat stiff surface, the lateral sheer stress ($F_{ss}$) can be expressed as^12^:

$F_{ss}\left( v \right)=\frac{24}{\pi^{3}}\gamma D\left( \cos\theta_{r}\left( v \right)-cos\theta_{a}\left( v \right) \right),$ (S14)

where *γ* represents the surface tension coefficient, 106 mN/m^13^ for potassium liquid. *D* denotes the diameter of the contact region. $\theta_{a}$ and $\theta_{r}$ stands for the dynamic advancing and receding contact angles and are highlighted in **Figure S12**, respectively. These two contact angles depend on the relative velocity *v* of the droplet with respect to substrate: as *v* increases, $\theta_{a}$ increases, whereas $\theta_{r}$ decreases. It thus can be inferred that $F_{ss}\propto$ *v*. In other words, for the process of **Figure 2c**, when *v* is sufficiently high, $F_{ss}$ can exceed the surface tension of the potassium droplet, triggering a crack in droplet’s surface and imitating a rupture in the oxide shell. Therefore, it is important that the potassium slips on a high-speed substrate. It is observed that in our optimized experimental condition with *v* reaching 12 m/s, the contact angles between the droplet and substrate can be measured in situ with the high-speed camera (**Figure S12**). $\theta_{a}$ is about 113° and $\theta_{r}$ is about 9°. It can be calculated that *F_ss_* reaches approximately 4.5 mN, significantly surpassing the droplet’s horizontal component of surface tension of 1.1 mN*cos (67°)=0.43 mN (characteristic droplet size ~1 cm), leading to the split of the potassium drop’s surface and thus ensuring the instantaneous homogeneous potassium film formation with oxidation effectively excluded.


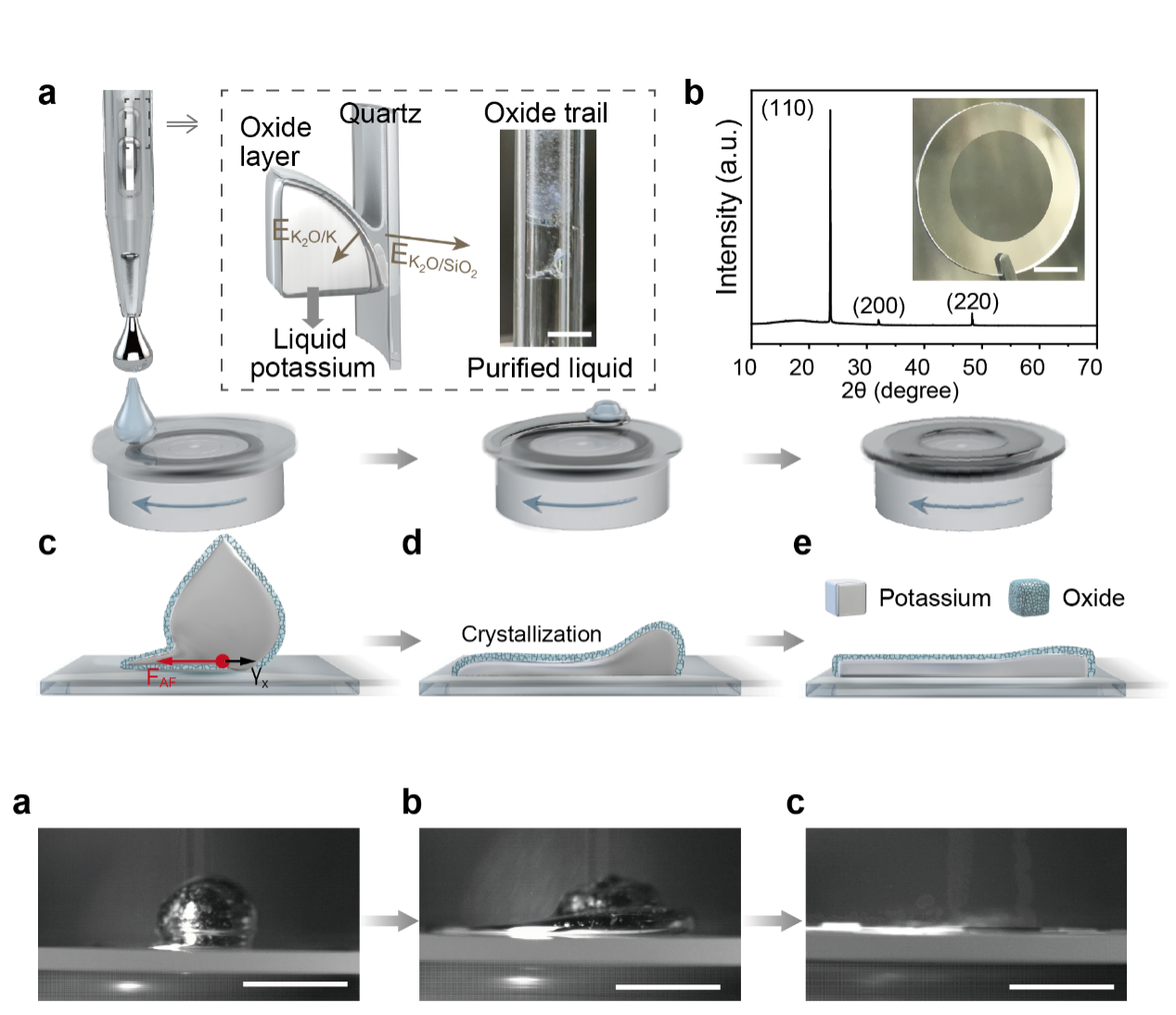


**Figure S11.** Optical images corresponding to the three main processes of crystallization process of SOC method, which taken by high-speed camera (scale bar: 5 mm).


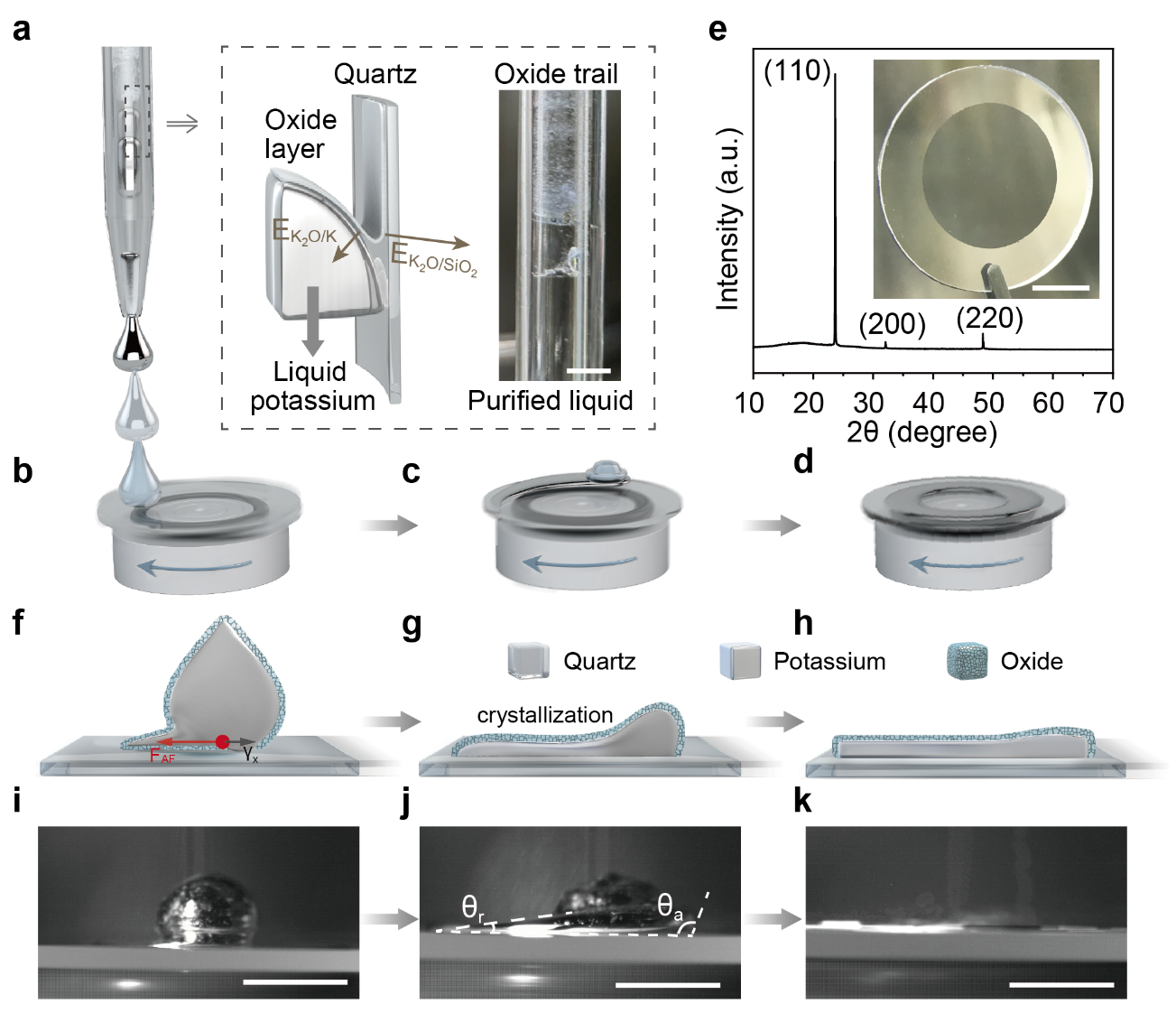


**Figure S12.** The highlighted $\theta_{a}$ and $\theta_{r}$ of slipping K droplet. Scale bar: 5 mm.

**S. Ⅱ. 4 Preparation process of ultra-smooth potassium samples for XRD tests**

The ultra-smooth potassium film samples for XRD tests are sealed with polyimide tape, an ionomer resin transparent to X-rays for a wide range of angles, and the process is shown in **Figure S13**.


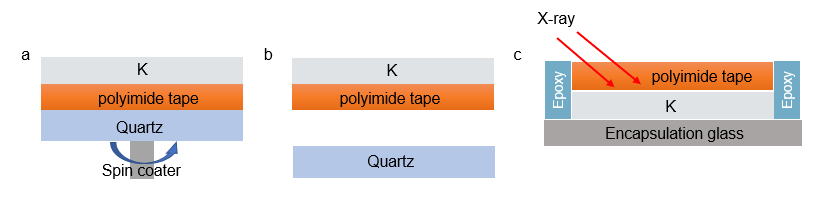


**Figure S13.** Preparation process of ultra-smooth potassium samples for XRD tests.

**S. Ⅱ. 5 Comparison of the crystalline nature of the potassium film prepared by SOC and sodium film fabricated by conventional thermo-assisted method**

**
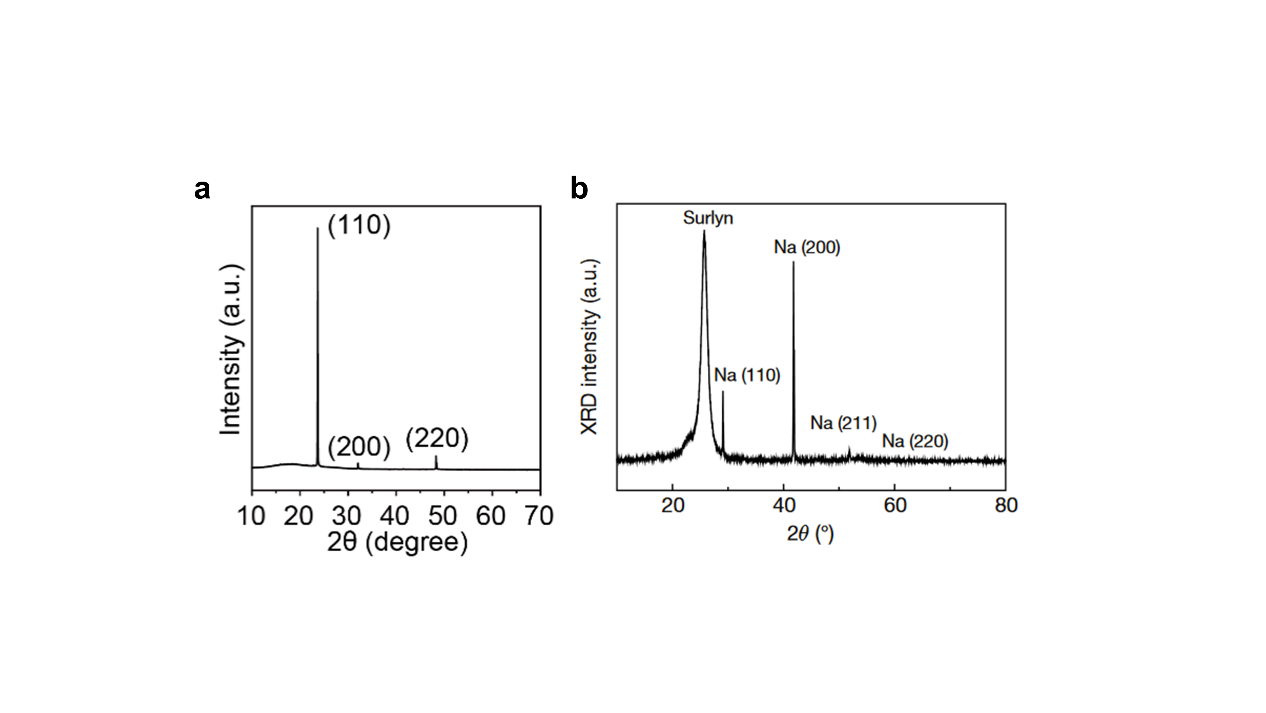
**

**Figure S14.** XRD characterizations on (a) the K film prepared by SOC and (b) Na film prepared by conventional thermo-assisted method reported in literature^11,14^.

**S. Ⅱ. 6** **Analysis of the optical image of the prepared potassium film**

**Figure S15** illustrates a typical K film prepared using our SOC method. The entire trajectory traversed by the K droplet is solidified into a smooth and uniform K film, corresponding to the process shown in **Figure 2c-e**. Notably, the white mark at the initial point represents an oxidation layer left by the initial contact between the freely falling K droplet and the substrate. During the subsequent slipping process, the liquid K directly contacts the substrate and solidifies into the film, without any interfacial inclusion of the oxide layer. The dark-field image shown in **Fig. S16** further confirms the high surface quality of the SOC-prepared K film, exhibiting a uniformly dark background with no pronounced localized scattering centers. Given that dark-field imaging is highly sensitive to surface roughness, grain boundaries, and nanoscale defects, the weak and spatially homogeneous scattering indicates the formation of a large-area, smooth metallic surface enabled by the SOC process.

**
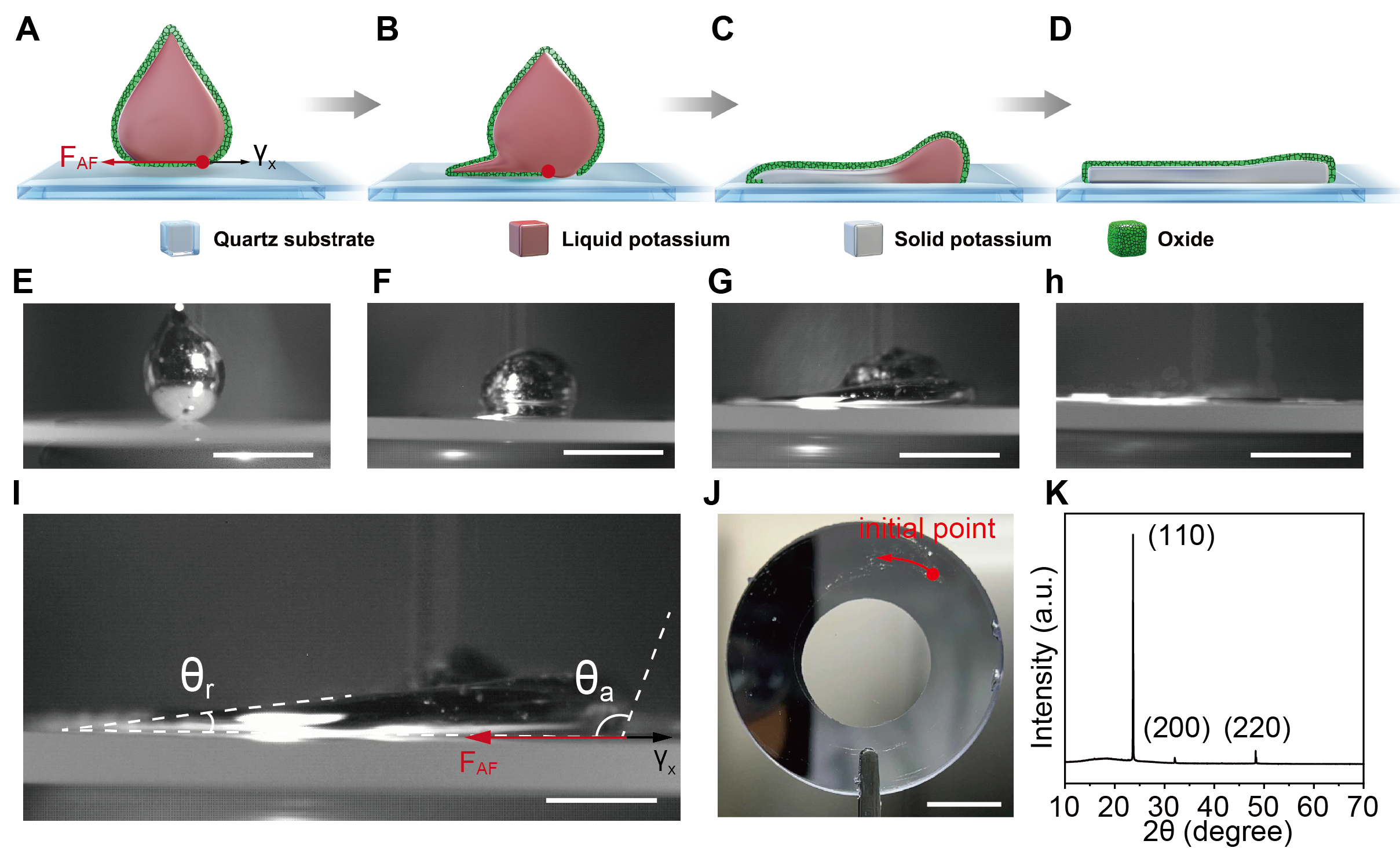
**

**Figure S15.** A typical K film prepared using the SOC method. The whole solidified potassium film along slipping trail is smooth while the initial point remains several oxidations (scale bar: 1cm).


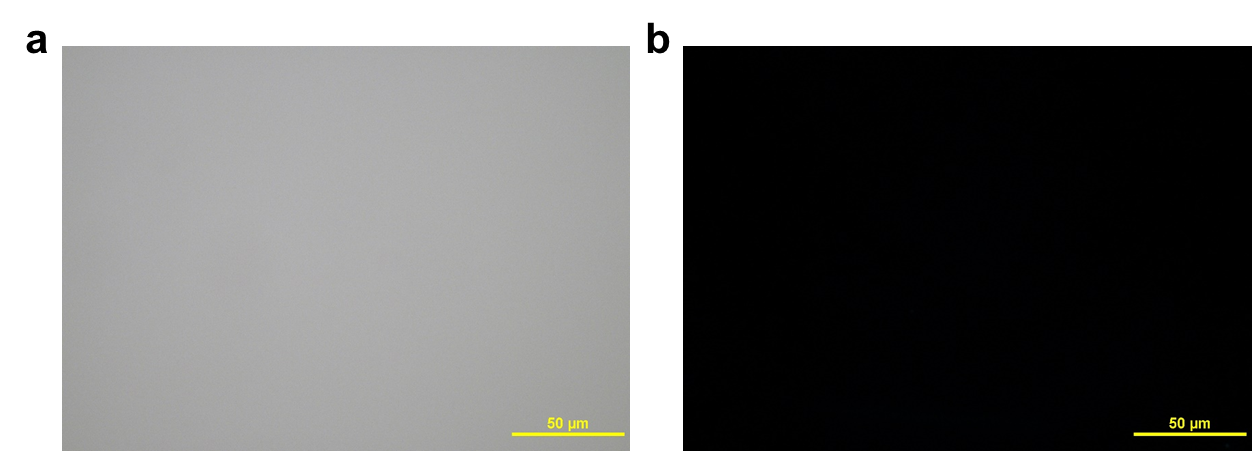


**Figure S16.** Bright-field (a) and dark-filed (b) images of the potassium film.

**S. II. 7 Dielectric function comparison of different materials**

**
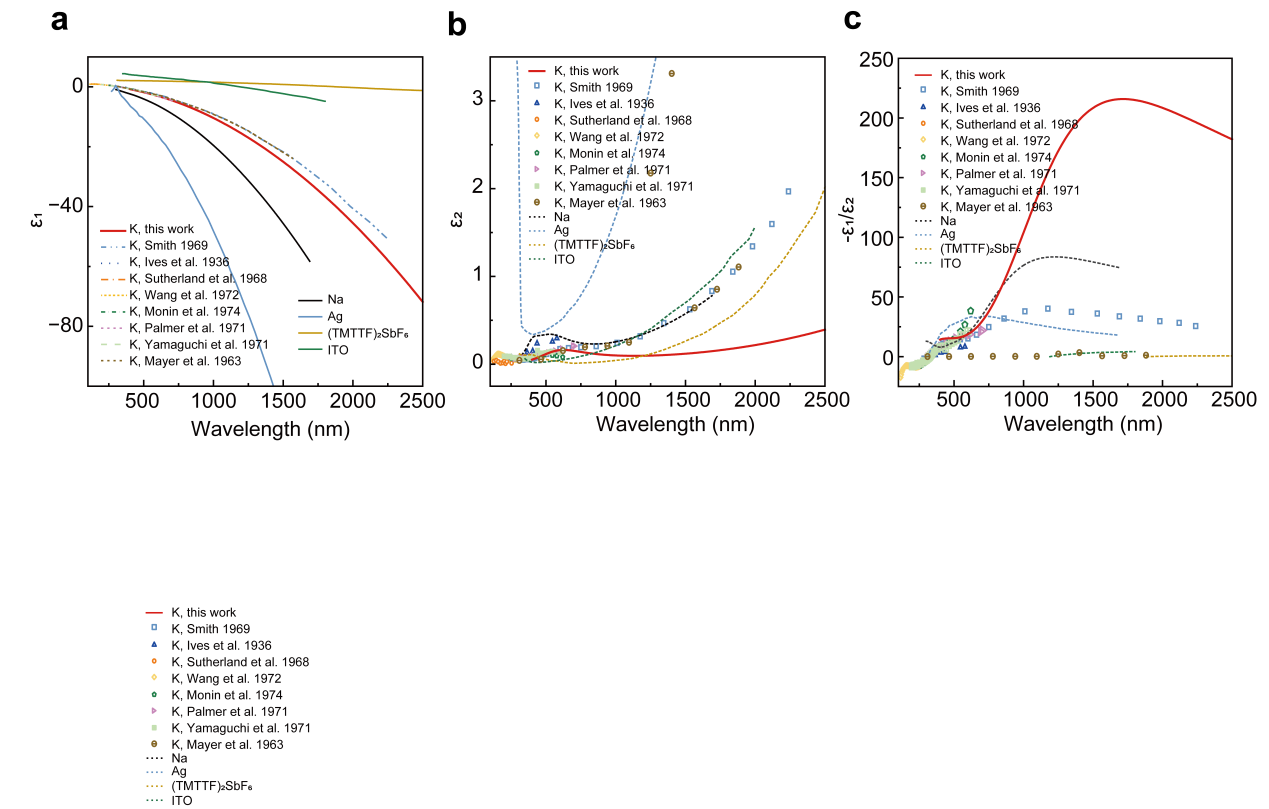
**

**Figure S17.** Dielectric functions of potassium thin films measured in this work and previously reported low-loss materials^11,15–24^.

**S. Ⅱ. 8 The fabrication process of the potassium-based plasmonic waveguide**

A 50-nm-thick silver film was first deposited on an ultrasmooth quartz substrate by electron-beam evaporation to serve as a conductive layer. In- and out-coupling gratings were then patterned on the silver film using focused ion beam (FIB) milling, with periods ranging from 750 to 900 nm and a duty cycle of approximately 1/2. Afterward, the silver film was selectively removed by etching in a nitric acid solution, yielding a flat quartz substrate with the grating structures preserved. Finally, a surface potassium layer was fabricated using the SOC method, resulting in a plasmonic waveguide structure (**Figure S18**).

To further visualize the cross-section of K/SiO_2_ coupling structure, the sample was transferred from the glove box to a vacuum chamber and then characterized by focused ion beam (FIB) milling, with the periodic grating profile clearly observed (marked by the yellow dashed line in Figure S20). Note that, due to the low melting point (63.65 °C), high-energy FIB operation without cold stage inevitably introduces partial melting and/or reconfiguration in potassium metal (see the emerging black hole area in **Figure S19d** compared **with 19c**); however, this does not affect the identification of the grating geometry used for SPP coupling.


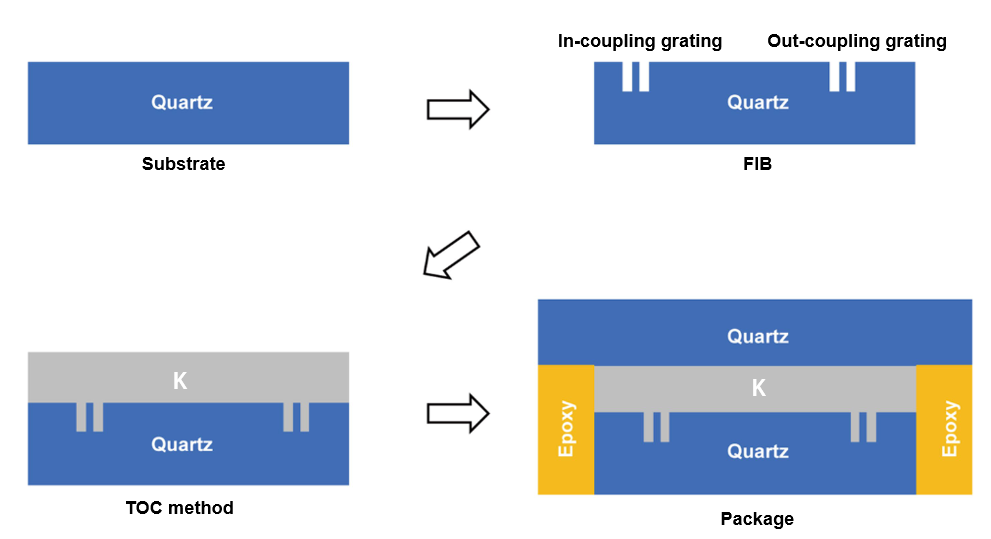


**Figure S18. Detailed fabrication process of the plasmonic waveguides.**

**
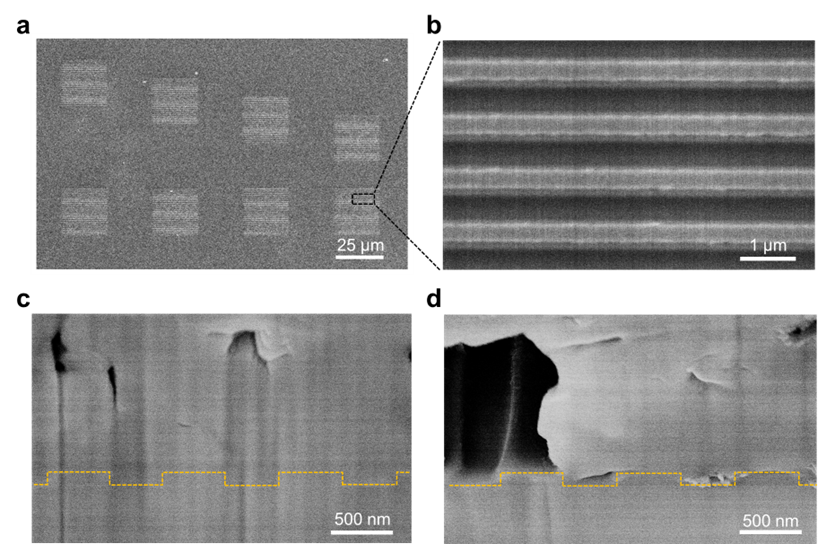
**

**Figure S19.** SEM images of the fabricated plasmonic waveguide structures. (a-b) Top-view of the 900 nm-period coupling grating on quartz substrate. (c-d) Cross-section of the same grating after K film fabricated using the SOC method.

**S. Ⅱ. 9** **Structure of semi-infinite SPP waveguide for measuring propagation length**

We fabricated potassium-based plasmonic waveguides in near-infrared to fit the optical loss from propagation length $L_{spp}$. The SPP mode in waveguides were excited through gratings. By varying period of grating (𝑃), we controlled the excitation wavelength of SPP, while keeping the fill factor at approximately 0.5 and the grating depth at around 150 nm. **Figure S20** shows the schematic (upper panel) and experimental (lower panel) configurations of the potassium-based plasmonic waveguide device, respectively. The left coupler (noted as downward red arrow) converts the incident laser beam to SPPs (left light spot), which propagate along the potassium–quartz interface and then out-coupled to free space via the right coupler (right light spot).


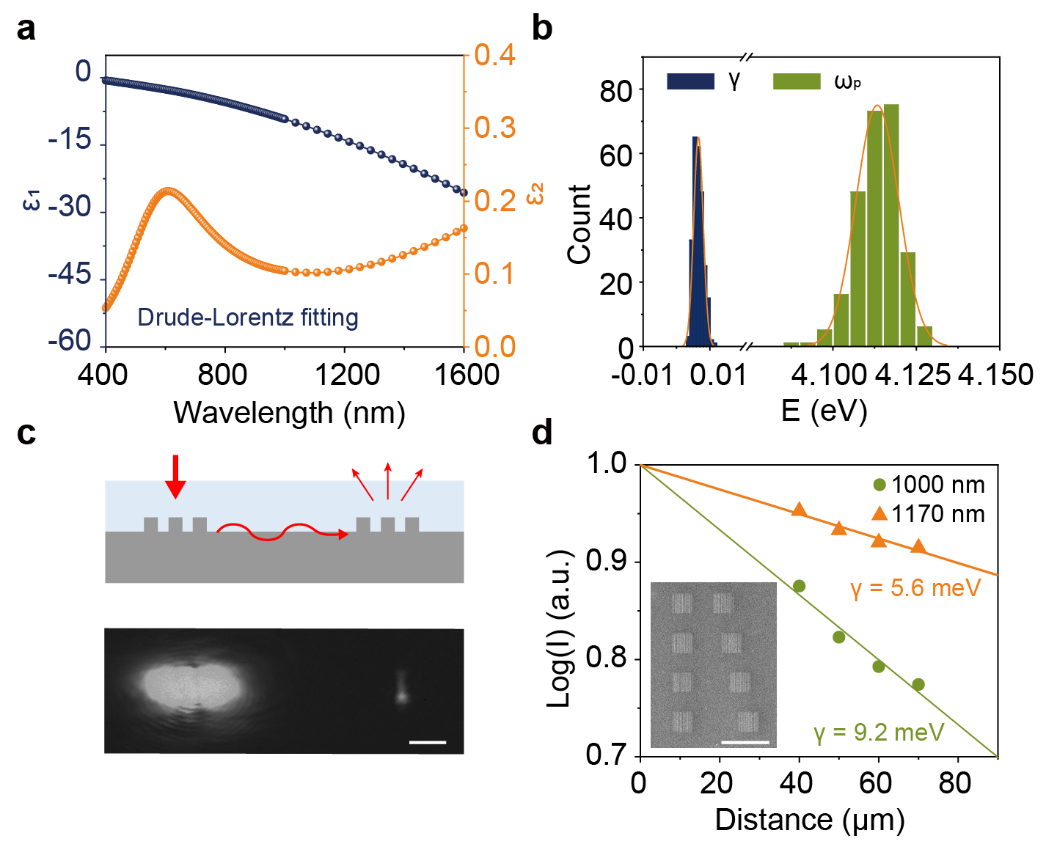


**Figure S20.** Schematic of potassium-based plasmonic waveguiding structure for propagation length measurement (up) and related optical image of the light spots (bottom). The scale bar is 20 μm.

**S. Ⅱ. 10** **The γ retrieved from propagation length and compared with sodium from previous literature**


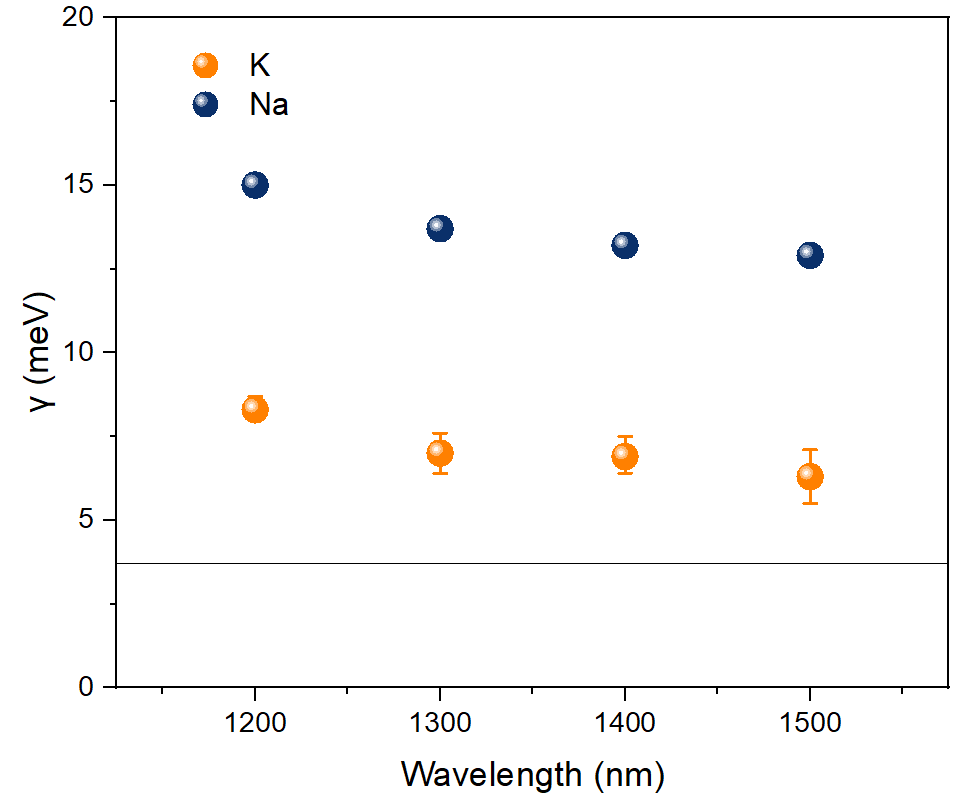


**Figure S21.** The magnified of γ retrieved from propagation length and compared with sodium from previous literature with error bar.

**S. Ⅱ. 11 Propagation measurements for potassium film with exponential curves fitted to the data**


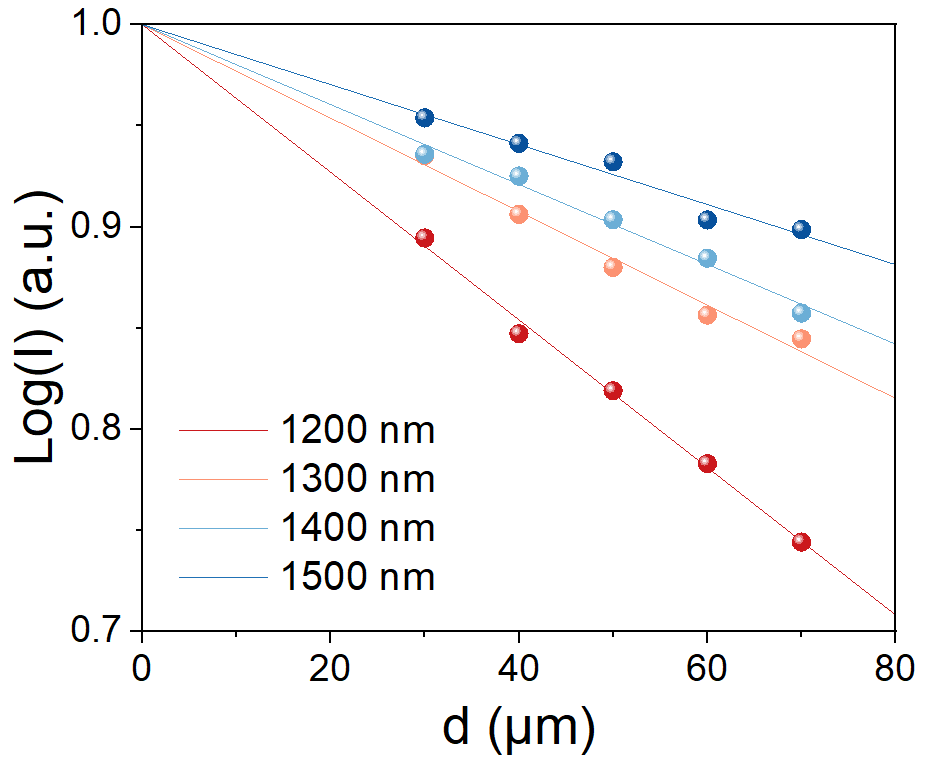


**Figure S22.** Propagation measurements at wavelengths of 1200 nm, 1300 nm, 1400 nm and 1500 nm for potassium film, with exponential curves fitted to the data.

**S. Ⅱ. 12 Optical loss extracted from propagation length through plasmonic waveguide device**

As the intensity of SPP decreases exponentially as a function of distance, we fitted the propagation length from various propagation separations with different distances as follow:

$lgI=-\frac{1}{L_{spp}}d+lgI_{0}$, (S15)

where *I* is the intensity of the outgoing light, *I_0_* is the intensity of the incident light and *d* is the distance between the two couplers. We then extracted optical loss *γ* from propagation length as follow:

Under the commonly used approximation $\varepsilon_{2}\ll\left| \varepsilon_{1} \right|$ that,

$L_{spp}=\frac{1}{2k_{I}}=\frac{\lambda}{2\pi\varepsilon_{1}\varepsilon_{2}}{[\frac{\varepsilon_{1}(\varepsilon_{1}+\varepsilon_{d})}{\varepsilon_{d}}]}^{\frac{3}{2}}$, (S16)

$\varepsilon_{1}=1-\frac{\omega_{p}^{2}}{\omega^{2}+\gamma^{2}}$, (S17)

$\varepsilon_{2}=\frac{\omega_{p}^{2}\gamma}{\omega(\omega^{2}+\gamma^{2})}$, (S18)

where $\varepsilon_{1}$ and $\varepsilon_{2}$ are the real and imaginary parts of the metal permittivity respectively, $\varepsilon_{d}$ is the dielectric constant of the quartz substrate, which is taken as 1.46, $\gamma$ is the fitted optical loss of potassium and the plasma frequency $\omega_{p}$ is generally related to the carrier concentration in the metal and is set to 4.0 eV. Because potassium exhibits negligible interband losses in the near-infrared regime, its dielectric function is intentionally described by a pure Drude model, without the inclusion of Lorentz oscillators.

**S. Ⅱ. 13** **Fabrication of metal/SiN/Air SPP device for s-SNOMs test**

We purchased TEM SiN grids (AR010C) with the grid size of 100 μm and the covered SiN thickness of ~ 40 nm. Using the TEM SiN grid as a substrate, we fabricated the K or Na film via the SOC process and Ag film via the physical vapor deposition (PVD) process, forming a metal/SiN/Air structure at the window. The 40 nm thick SiN layer ensures that oxygen do not come into contact with the metal film, while also providing a measurement window for s-SNOM testing.

**S. Ⅱ. 14 Real-space fringe profiles and corresponding FT profiles of the s-SNOM imaging data**


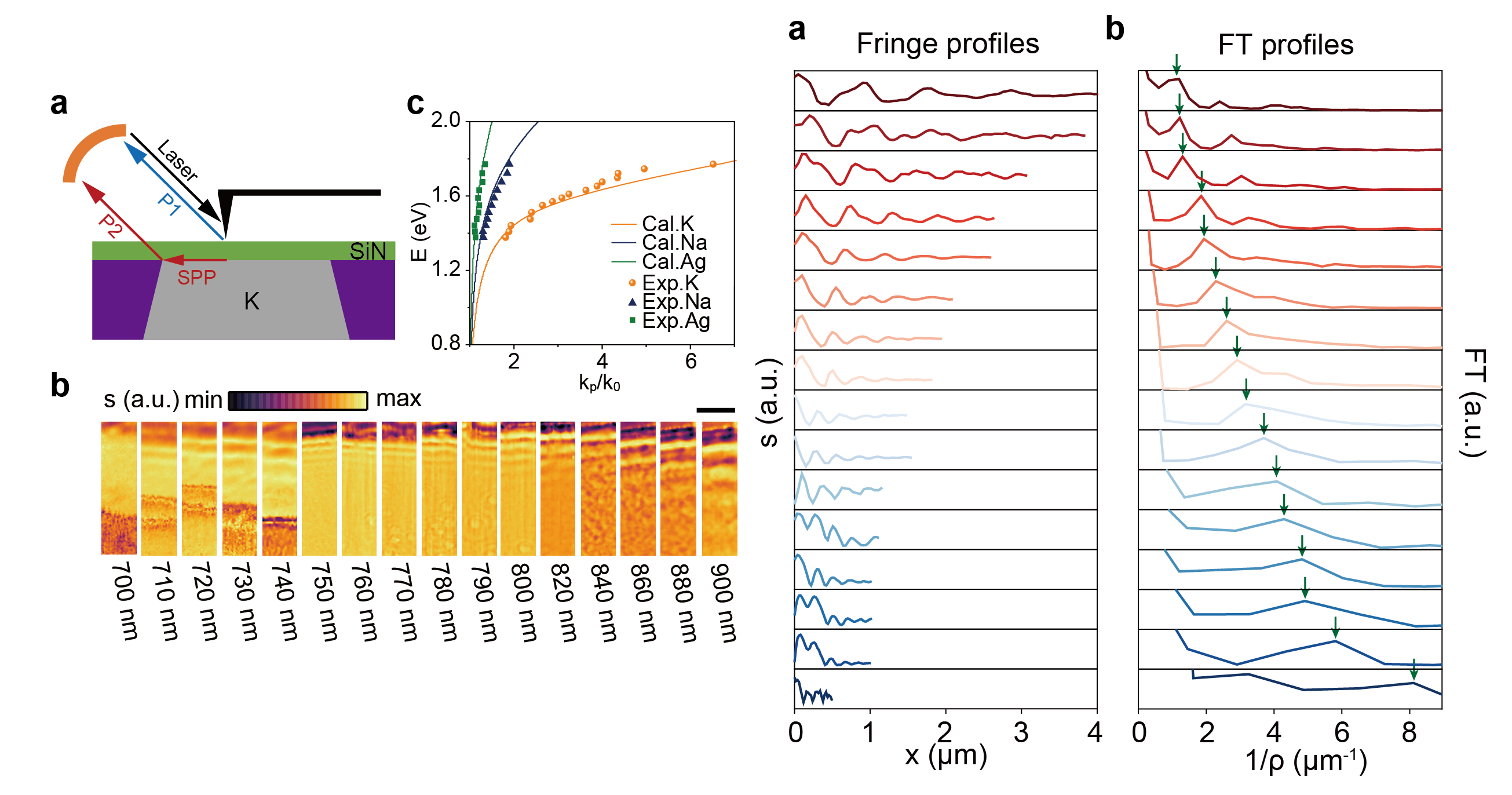


**Figure S23.** Real-space fringe profiles (a) and corresponding FT profiles (b) of the s-SNOM imaging data of Fig. 4(b).

**S. Ⅱ. 15 Time-dependent measurements for stability characterization**


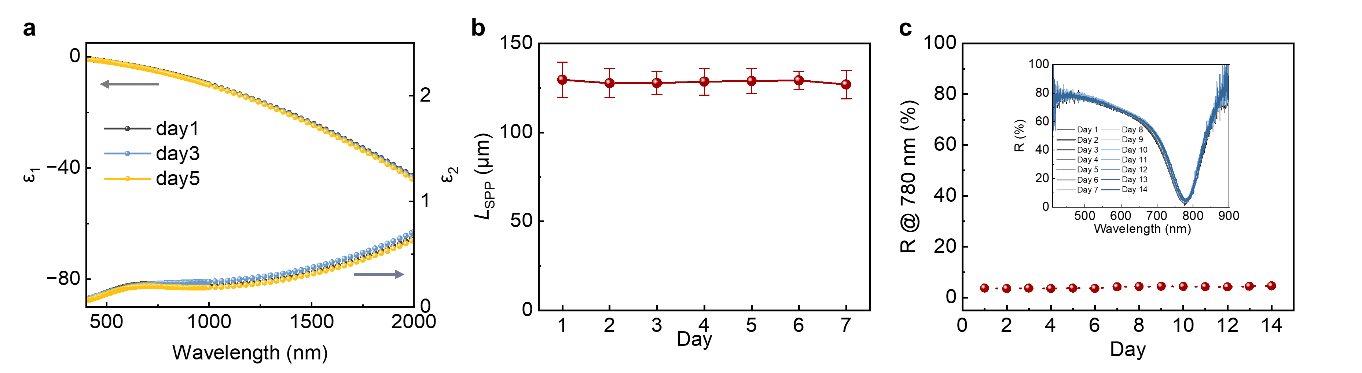


**Figure S24.** (a) Temporal stability of the dielectric functions extracted from spectroscopic ellipsometry. (b) Temporal stability of the SPP propagation length (*L_SPP_*) measured at 1400 nm on the K/quartz interface. (c) Temporal stability of the reflectance spectra of a nanostructured K film fabricated on a silica substrate patterned with nanoholes (period: 250 nm, diameter: 230 nm, depth: 60 nm).

**Reference**

1. Albers, R. C., Bohlin, L., Roy, M. & Wilkins, J. W. Normal and umklapp phonon decay rates due to phonon-phonon and electron-phonon scattering in potassium at low temperatures. *Phys. Rev. B* **13**, 768–786 (1976).

2. Lawrence, W. & Wilkins, J. Umklapp electron-phonon scattering in the low-temperature resistivity of polyvalent metals. *Phys. Rev. B* **6**, 4466 (1972).

3. Trofimenkoff, P. N. & Ekin, J. W. Electron-Phonon Umklapp Scattering Processes in the Low-Temperature Ultrasonic Attenuation and Electrical Resistivity of Potassium. *Phys. Rev. B* **4**, 2392–2397 (1971).

4. Wagner, D. & Bowers, R. The radio-frequency size effect: A tool for the investigation of conduction electron scattering in metals. *Adv. Phys.* **27**, 651–746 (1978).

5. Gasparov, V. A. & Huguenin, R. Electron-phonon, electron-electron and electron-surface scattering in metals from ballistic effects. *Advances in Physics* **42**, 393–521 (1993).

6. Gasparov, V. & Huguenin, R. Electron-phonon, electron-electron and electron-surface scattering in metals from ballistic effects. *Adv. Phys.* **42**, 393–521 (1993).

7. Bardeen, J. & Shockley, W. Deformation potentials and mobilities in non-polar crystals. *Phys. Rev.* **80**, 72 (1950).

8. Khan, F. & Allen, P. Deformation potentials and electron-phonon scattering: Two new theorems. *Phys. Rev. B* **29**, 3341 (1984).

9. Lawrence, W. Electron-electron scattering in the low-temperature resistivity of the noble metals. *Phys. Rev. B* **13**, 5316 (1976).

10. Khurgin, J. B. Ultimate limit of field confinement by surface plasmon polaritons. *Faraday Discuss.* **178**, 109–122 (2015).

11. Wang, Y. *et al.* Stable, high-performance sodium-based plasmonic devices in the near infrared. *Nature* **581**, 401–405 (2020).

12. Pilat, D. W. *et al.* Dynamic Measurement of the Force Required to Move a Liquid Drop on a Solid Surface. *Langmuir* **28**, 16812–16820 (2012).

13. Jordan, D. & Lane, J. The surface tension of liquid sodium and liquid potassium. *Aust. J. Chem.* **18**, 1711 (1965).

14. Gao, Z. *et al.* Low-loss plasmonics with nanostructured potassium and sodium–potassium liquid alloys. *Nano Lett.* **23**, 7150–7156 (2023).

15. Ives, H. E. & Briggs, H. B. The Optical Constants of Potassium. *J. Opt. Soc. Am.* **26**, 238 (1936).

16. Sutherland, J. C. & Arakawa, E. T. Optical Properties of Potassium for Photons of Energy 396 to 969 eV*. *J. Opt. Soc. Am.* **58**, 1080 (1968).

17. Naby, M. H. E. Die optischen Konstanten des Kaliums im Wellenliingenbereich von 3650 bis 20000. Z. Physik **174**, 269 (1963).

18. Smith, N. V. Optical Constants of Sodium and Potassium from 0.5 to 4.0 eV by Split-Beam Ellipsometry. *Phys. Rev.* **183**, 634–644 (1969).

19. Smith, N. V. Observation of surface plasmons and measurement of the optical constants for sodium and potassium. *Phys. Rev.* **183**, 634 (1969).

20. Whang, U. S., Arakawa, E. T. & Callcott, T. A. Optical Properties of K between 4 and 10.7 eV and Comparison with Na, Rb, and Cs. *Phys. Rev. B* **6**, 2109–2118 (1972).

21. Monin, J. & Boutry, G.-A. Optical and photoelectric properties of alkali metals. *Phys. Rev. B* **9**, 1309–1327 (1974).

22. Wu, Z. *et al.* Hyper-gap transparent conductor. *Nat. Mater.* **24**, 1387–1392 (2025).

23. Yang, H. U. *et al.* Optical dielectric function of silver. *Phys. Rev. B* **91**, 235137 (2015).

24. Yamaguchi, S. and Hanyu, T. Optical properties of potassium. *Journal of the Physical Society of Japan* **31**, 1431–1441 (1971).
